# Strawberry Flavor: Diverse Chemical Compositions, a Seasonal Influence, and Effects on Sensory Perception

**DOI:** 10.1371/journal.pone.0088446

**Published:** 2014-02-11

**Authors:** Michael L. Schwieterman, Thomas A. Colquhoun, Elizabeth A. Jaworski, Linda M. Bartoshuk, Jessica L. Gilbert, Denise M. Tieman, Asli Z. Odabasi, Howard R. Moskowitz, Kevin M. Folta, Harry J. Klee, Charles A. Sims, Vance M. Whitaker, David G. Clark

**Affiliations:** 1 Plant Molecular and Cellular Biology Program, University of Florida, Gainesville, Florida, United States of America; 2 Department of Environmental Horticulture, University of Florida, Gainesville, Florida, United States of America; 3 College of Dentistry, University of Florida, Gainesville, Florida, United States of America; 4 Horticultural Sciences Department, University of Florida, Gainesville, Florida, United States of America; 5 Food Science and Human Nutrition Department, University of Florida, Gainesville, Florida, United States of America; 6 Moskowitz Jacobs Inc., White Plains, New York, United States of America; 7 Gulf Coast Research and Education Center, University of Florida, Wimauma, Florida, United States of America; 8 Plant Innovation Program, University of Florida, Gainesville, Florida, United States of America; RIKEN PSC Japan

## Abstract

Fresh strawberries (*Fragaria x ananassa*) are valued for their characteristic red color, juicy texture, distinct aroma, and sweet fruity flavor. In this study, genetic and environmentally induced variation is exploited to capture biochemically diverse strawberry fruit for metabolite profiling and consumer rating. Analyses identify fruit attributes influencing hedonics and sensory perception of strawberry fruit using a psychophysics approach. Sweetness intensity, flavor intensity, and texture liking are dependent on sugar concentrations, specific volatile compounds, and fruit firmness, respectively. Overall liking is most greatly influenced by sweetness and strawberry flavor intensity, which are undermined by environmental pressures that reduce sucrose and total volatile content. The volatile profiles among commercial strawberry varieties are complex and distinct, but a list of perceptually impactful compounds from the larger mixture is better defined. Particular esters, terpenes, and furans have the most significant fits to strawberry flavor intensity. In total, thirty-one volatile compounds are found to be significantly correlated to strawberry flavor intensity, only one of them negatively. Further analysis identifies individual volatile compounds that have an enhancing effect on perceived sweetness intensity of fruit independent of sugar content. These findings allow for consumer influence in the breeding of more desirable fruits and vegetables. Also, this approach garners insights into fruit metabolomics, flavor chemistry, and a paradigm for enhancing liking of natural or processed products.

## Introduction

Modern fully ripe strawberry (*Fragaria x ananassa)* fruit is characterized by its large size [1], vibrant red color [2], reduced firmness [3], distinct aroma [4], and sweet fruity flavor [5]. The flesh of the strawberry is a swollen receptacle, a false fruit, and the seeds or achenes are the true fruit [6], which will be collectively referred to as strawberry fruit. The three stages of non-climacteric, auxin dependent strawberry fruit development; division, expansion and ripening, involve gains in diameter and fresh weight; during which color shifts from green to white to dark red in roughly forty days following anthesis [7]. Ripening of strawberry fruit results in the accumulation of multiple sugars and organic acids, culminating with peak volatile emission [8].

Flavor is the perceptual and hedonic response to the synthesis of sensory signals of taste, odor, and tactile sensation [9]. In the case of strawberry and other fruits, sensory elicitation is the result of multiple direct interactions between plant and human: sugars and acids, pigments, turgor and structure, and volatile compounds, which elicit the senses of taste, vision, tactile sensation, and olfaction, respectively, in the development of flavor [10]–[13]. A consumer based survey indicated sweetness and complex flavor as consistent favorable attributes of the “ideal” strawberry experience [14]. Much emphasis is placed on sugars, acids, and volatile compounds as these metabolites are primary sensory elicitors of taste and olfaction which attenuate the perception and hedonics of sweetness and flavor. Thus a ripe strawberry is metabolically poised to elicit the greatest sensory and hedonic responses from consumers.

During strawberry fruit development sucrose is continually imported from photosynthetic tissue. A consistently high sucrose invertase activity contributes to carbon sink strength in all developmental stages of fruit [15]. Delivered sucrose is hydrolyzed into glucose and fructose, and these three carbohydrates constitute the major soluble sugars of ripe strawberries, a result of their continual accumulation during fruit development [16]. In fact, an approximately 150% increase in their sum during ripening has been observed [8], [15]. The influx of carbon initiates a complex network of primary and secondary metabolism specific to ripening strawberry fruit [16]. For example, the metabolic activity of ripening strawberry is visualized by the late accumulation of the predominant red pigment, pelargonidin 3-glucoside [17], an anthocyanin derived from the primary metabolite phenylalanine [16].

The dynamics of fruit development are genetically driven. Microarray analysis determined nearly 15% of probed expressed genes exhibit significant differential expression (60% up, 40% down) in red compared to green fruit [18]. One up regulated gene, *Polygalacturonase 1* (*FaPG1*), contributes to fruit softening [19] by aiding in catalytic cell wall disassembly [20]. Reduction of firmness is also attributed to dissolution of middle lamella, a pectin rich cell wall layer that functions in cell-to-cell adhesion [3]. Active shifts in transcription throughout ripening result in metabolic network reconfiguration altering the chemical and physical properties.

Metabolic profiling indicates an accumulation of sugars, organic acids, and fatty acids as well as the consumption of amino acids during fruit development. Subsequently alkanes, alcohols, aldehydes, anthocyanins, ketones, esters, and furanones increase during fruit ripening [7]. Many of these chemical classes serve as precursors to volatile synthesis [21], thus facilitating a metabolic flux through biosynthetic pathways for increased and diverse volatile emissions in ripe strawberry fruit, predominantly furans, acids, esters, lactones, and terpenes [8]. Over 350 volatile compounds have been identified across *Fragaria* [22], however within a single fruit, far fewer compounds are detectable and even less contribute to aroma perception.

A cross comparison of five previous studies which analyze strawberry volatiles depicts the lack of agreement in defining chemical constituents of strawberry aroma. Each source considers a highly variable subset of volatiles, which are determined by signal intensity and/or human perception of separated compounds [4], [5], [23]–[25]. Mutual volatiles across studies include butanoic acid, methyl ester; butanoic acid, ethyl ester; hexanoic acid, methyl ester; hexanoic acid, ethyl ester; 1,6-octadien-3-ol, 3,7-dimethyl- (linalool); butanoic acid, 2-methyl-; and 3(2*H*)-furanone, 4-methoxy-2,5-dimethyl-, the current consensus of integral strawberry aroma compounds. Comparisons of consumer preference among a variety of fresh strawberries and their volatile profiles describes less preferable varieties as possessing less esters, more decalactones and hexanoic acid [4]. The breadth of volatile phenotypes previously reported highlights the diversity across strawberry genotypes and underscores the complexity of the aggregate traits of aroma and flavor.

Florida strawberry production is concentrated on ten thousand acres near the Tampa Bay. Mild winters allow for annual horticulture which requires continual harvest of ripe fruit from late November through March. Environmental effects on fruit quality are partially attributed to gradually increasing temperatures beginning in mid-January. One result is a late season decline of soluble solids content (SSC) [26], [27]. In fact, increasing temperature is known to be responsible for increasing fruit maturation rate and decreasing SSC independent of flowering date [26]. Previous work also identifies variability of SSC, as well as titratable acidity (TA) and multiple classes of volatile compounds across harvest dates [28]. The complex fruit biochemistry, which is variably affected by genetic, environmental, and developmental factors, coupled with individuals’ perceptional biases has made defining strawberry flavor cumbersome.

**Figure 1:**
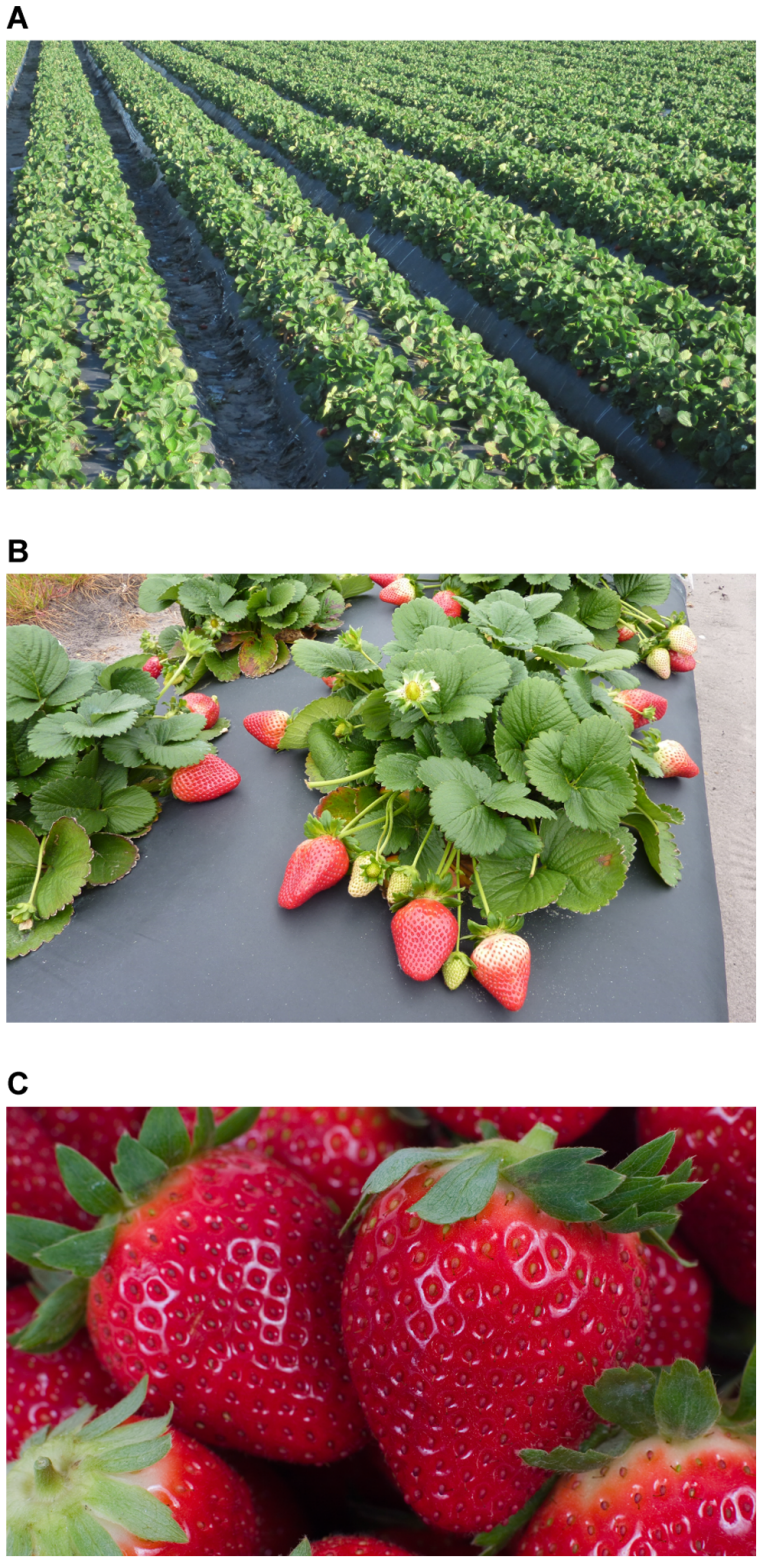
Photographs of strawberry production field, plants, and harvested fruits. Photographs characterizing the commercial style production and harvest standards employed in this study. Annual plasticulture of strawberry (A) is common practice in Florida production fields. Winterstar™ strawberry plants (B) bearing flowers and fruit of varying developmental stages and ripeness. Harvested fruit of cultivar ‘Winter Dawn’ (C) demonstrating ripeness used in study, 90–100% red.

Here we exploit the genetic and within-season variability of fruit to provide as many unique strawberry experiences as possible to a large sample of consumers. To enhance the range and diversity of flavors and chemical constituents 35 genetic backgrounds were included: public and private cultivars representing a large proportion of commercial strawberry acreage in North America, University of Florida advanced breeding selections, and European cultivars (Fig. 1). Parallel assays of ripe strawberry samples quantify fruit traits of TA, pH, and fruit firmness, as well as the content of malic acid, citric acid, glucose, fructose, sucrose, and 81 volatile compounds of diverse chemical classes. The contributions of these attributes to fruit quality is determined by simultaneously evaluating samples for perceived sensory intensities of sourness, sweetness, and strawberry flavor, as well as the hedonic responses of texture liking and overall liking by consumer panelists. Data analyses determine significant biochemical and consumer response differences between early and late season fruit, gross variation of strawberry experiences, and factors influencing hedonics and sensory perception of strawberry fruit consumption using a psychophysics approach. Ultimately, an effect of particular volatile constituents to enhance sweetness intensity independent of sugar content of fruit was found. These findings have great implications in the breeding of more desirable fruits and vegetables, as well as for the food industry as a whole.

## Methods

### Ethics Statement

All human consumer panels are conducted at the Food Science and Human Nutrition Department at the University of Florida in Gainesville, FL. The University of Florida Institutional Review Board 2 (IRB2) chaired by Ira S. Fischler approved the protocol and written consent form (case 2003-U-0491), which participants are required to complete.

### Plant Material

Thirty-five strawberry cultivars and selections were grown at or in the near vicinity of the Gulf Coast Research and Education Center (14625 County Road 672, Wimauma, FL) during the 2010–2011 (season 1) and 2011–2012 (season 2) winter seasons. Fruit are cultivated according to current commercial practices for annual strawberry plasticulture in Florida [1], [29](Fig. 1A). The cultivars are chosen to represent a large proportion of commercial strawberry acreage in North America from both public and private breeding programs. Additional breeding selections and European cultivars are added to enhance the range of diversity for flavors and chemical constituents. Weekly cultivar representation is determined by fruit availability during a particular harvest week and attempting to maximize genetic diversity, except for the highly replicated cultivar ‘Festival’. Fully-ripe fruit by commercial standards, 90–100% red compared to white [30] (Fig. 1B-C), is harvested from three to five cultivars on Monday mornings, delivered to the respective laboratories, and stored at 4°C in the dark overnight for simultaneous analysis of fresh strawberry fruit volatiles, firmness, and sensory analysis on Tuesdays; as well as sample preparation for later sugar and acid measurements. Six harvests in both seasons allows for the complete analysis of 54 samples. Weather data is obtained from the Balm, FL station of the Florida Automated Weather Network (http://fawn.ifas.ufl.edu/data/reports) for date ranges January 3, 2011 through February 28, 2011 and December 26, 2011 through March 13, 2012. Daily maximum and minimum temperature recording height is 60 cm, and daily average relative humidity, rainfall, and solar radiation are recorded at 2 m.

### Volatile Analysis

At least 100 g or seven berries of each sample are removed from 4°C dark overnight storage prior to volatile collection. Samples are homogenized in a blender prior to splitting into three 15 g replicates for immediate capturing of volatile emissions. The remainder is frozen in N_2_ (l) and stored at –80°C for later sugar and acid quantification. A two hour collection in a dynamic headspace volatile collection system [31] allows for concentration of emitted volatiles on HaySep 80–100 porous polymer adsorbent (Hayes Separations Inc., Bandera, TX, USA). Elution from polymer is described by Schmelz [32].

Quantification of volatiles in an elution is performed on an Agilent 7890A Series gas chromatograph (GC) (carrier gas; He at 3.99 ml min^−1^; splitless injector, temperature 220°C, injection volume 2 µl) equipped with a DB-5 column ((5%-Phenyl)-methylpolysiloxane, 30 m length ×250 µm i.d. × 1 µm film thickness; Agilent Technologies, Santa Clara, CA, USA). Oven temperature is programmed from 40°C (0.5 min hold) at 5°C min^−1^ to 250°C (4 min hold). Signals are captured with a flame ionization detector (FID) at 280°C. Peaks from FID signal are integrated manually with Chemstation B.04.01 software (Agilent Technologies, Santa Clara, CA). Volatile emissions (ng^1^ gFW^−1^ h^−1^) are calculated based on individual peak area relative to sample elution standard peak area. GC-mass spectrometry (MS) analysis of elutions are performed on an Agilent 6890N GC in tandem with an Agilent 5975 MS (Agilent Technologies, Santa Clara, CA, USA) and retention times are compared with authentic standards (Sigma Aldrich, St Louis, MO, USA) for volatile identification [33]. Chemical Abstract Services (CAS) registry numbers were used to query SciFinder® substances database for associated chemical name and molecular formula presented in Table S1.

### Sugars and Acids Quantification

Titratable acidity, pH, and soluble solids content [26] are averaged from four replicates of the supernatant of centrifuged thawed homogenates [1]. An appropriate dilution of the supernatant from a separate homogenate (centrifugation of 1.5 ml at 16,000 x *g* for 20 min) is analyzed using biochemical kits (per manufacturer’s instructions) for quantification of citric acid, L-malic acid, D-glucose, D-fructose, and sucrose (CAT# 10-139-076-035, CAT# 10-139-068-035, and CAT# 10-716-260-035; R-Biopharm, Darmstadt, Germany) with absorbance measured at 365 nm on an Epoch Microplate Spectrophotometer (BioTek, Winooksi, VT, USA). Metabolite average concentration (mg^1^ 100 gFW^−1^) is determined from two to six technical replicates per pooled sample. Derived sucrose concentrations via D-glucose and D-fructose are mathematically pooled.

### Firmness Determination

Firmness of the strawberries is determined as the resistance of the fruit to penetration at its equator with a TA.XTPlus Texture Analyzer (Texture Technologies Corp., Scarsdale, NY, USA; Stable Micro Systems, Godalming, Surrey, UK). The Texture Analyzer is equipped with a 50 kg load cell and an 8 mm diameter convex tip probe. Whole fruit is penetrated on the side to 7 mm down from the epidermis at a test speed of 2 mm^1^ sec^−1^; a flap cut off the opposite provides stability. Maximum force in kg for eight fruit is averaged and reported as a measure of firmness.

### Sensory Analysis

Over the course of two annual seasons, 166 recruited strawberry consumers (58 male, 108 female) evaluate strawberry cultivars. Ages of panelist ranged from 18 to 71, with a median age of 24. Panelists self-classified themselves as 98 White or Caucasian, 11 Black or African-American, 1 Native American, Alaska Native or Aleutian, 41 Asian/Pacific Islander, and 15 Other. An average of 106 (range of 98–113) panelists evaluated between three and five cultivars per session [34]. Fresh, fully-ripe strawberry fruit is removed from overnight 4°C dark storage and allowed to warm to room temperature prior to sensory analysis. Each panelist is given one to two whole strawberries for evaluation, depending on cultivar availability. Panelists bite each sample, chew, and swallow it. Ratings for overall liking and texture liking are scaled on hedonic general labeled magnitude scale (gLMS) from –100 to +100, *i.e.* least to most pleasurable experience [34]–[37]. Perceived intensity of sweetness, sourness, and strawberry flavor are scaled in context of all sensory experiences using sensory gLMS that ranges from 0 to +100, *i.e.* none to most intense sensory stimulus [34]–[37]. Scales are employed to mediate valid comparisons across subjects and sessions.

### Statistical Analysis

Means and standard errors for consumer, physical, and metabolite measurements are determined from all replicates using JMP (Version 8, SAS Institute Inc., Cary, NC, USA). One-way analysis of early and late season fruit quality and consumer response measures was subjected to mean comparison using Tukey’s HSD (α = 0.05). Bivariate analysis among individual measurements of samples allows for linear fit, which includes summary of fit, analysis of variance, t-test, and correlation analysis for density ellipse. Two-way Ward hierarchical cluster analysis of all quantified metabolite and strawberry samples is accomplished in JMP. Amounts of individual volatile compounds are regressed using the “enter” method in SPSS (IBM Corp., Armonk, NY, USA). This is done individually for each of the three sugars: glucose, fructose or sucrose to identify which compounds have an effect on sweetness intensity [14] independent of each of the sugars. For *p*-values ≤ 0.05, the volatile makes a contribution to perceived sweetness that is independent of the sugar tested.

## Results

**Figure 2:**
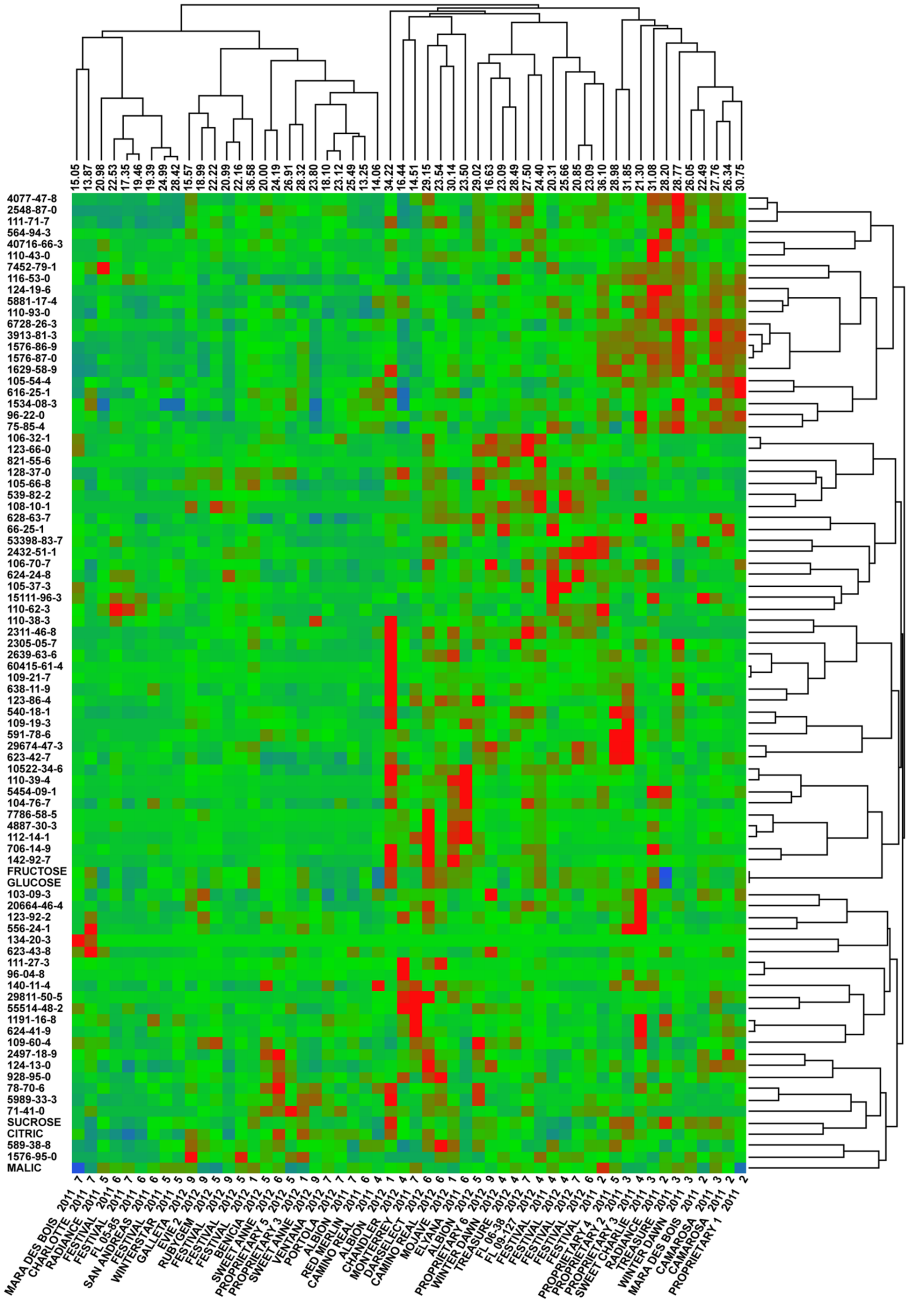
Cluster analysis of strawberry samples and quantified metabolites. Two-way Ward cluster analysis of strawberry samples (bottom) and quantified single metabolites (right) with overall liking score of sample (top) constructed using JMP 8. Standardization of metabolite content is by row mean and standard deviation, with high values represented as red, average as green, and low as blue. The hierarchy and distance of segments within the vertical dendrogram indicates relatedness of content across samples for single metabolites. Structure of the horizontal dendrogram indicates relatedness of all metabolite contents among individual samples.

The inventory of 54 fully ripe (Fig. 1C) unique strawberry samples (35 cultivars, 12 harvests, two seasons) assayed for TA, pH, firmness, as well as the concentrations of malic acid, citric acid, glucose, fructose, sucrose, and quantity of 81 volatile compounds is reported (Table S2). Cluster analysis of relative chemical composition of all samples and derived hierarchy of both cultivar and metabolite relatedness is displayed (Fig. 2). The vertical dendrogram (Fig. 2) demonstrates the lack of relatedness among volatile compound quantities through large distances of initial segments, as well as the high number of clusters. Slightly more structure is observed among the samples, horizontal dendrogram (Fig. 2), due to genetic or environmental effects.

### Progression of Harvest Season Affects Perceived Quality and Metabolite Content of Strawberry

Overall liking is a measure of pleasure derived from consuming a strawberry sample. The two samples with the greatest overall liking ratings are of cultivar ‘Festival’. Fruit harvested early in the season, week 2 of season 1 and week 1 of season 2, elicit overall likings of 36.1 and 36.6, respectively (Table 1). Five weeks following both early samplings of ‘Festival’ the overall liking of the same cultivar decreases below the sample set median of 23.5 (Table S2) to 17.3 in season 1 week 7 and to 23.1 in season 2 week 6 (Table 1). Therefore the earlier season samples elicit a greater hedonic response than late season samples. Overall likings are determined using the hedonic general labeled magnitude scale that ranges from –100 to +100, *i.e.* least to most pleasurable experience [34]–[37]. Conversely, sweetness, sourness, and strawberry flavor are measured using the sensory intensity general labeled magnitude scale that ranges from 0 to +100, *i.e.* none to most intense sensory stimulus [34]–[37]. Consumer perception of sweetness and strawberry flavor intensity decrease significantly between the same pairs of early and late season ‘Festival’ fruit (Table 1). Significant biochemical differences between early and late samples include decreased content of glucose, fructose, sucrose, and total volatiles. The early ‘Festival’ from the first season contains 88% more total sugar and 65% more total volatiles than the late ‘Festival’ of the same season (Table 1), demonstrating the disparity between early and late harvest week fruit quality and its effect on consumer sensory perception and acceptability.

**Table 1:** Comparison of early and late season strawberry fruit.

			Season 1				Season 2			
			Week 2		Week 7		Week 1		Week 6	
Mean week temperature										
	Daily maximum	°C	21.6	B	28.2	A	21.3	B	26.1	A
	Daily minimum	°C	7.4	B	13.3	A	6.7	B	13.1	A
	Daily average	°C	14.9	B	20.3	A	14.0	B	19.0	A
Consumer ratings										
	Overall liking	–100 to +100	36.1	A	17.3	B	36.6	A	23.1	B
	Texture liking	–100 to +100	35.7	A	23.8	B	34.8	A	24.3	B
	Sweetness intensity	0 to +100	30.3	A	15.9	B	34.0	A	22.2	B
	Sourness intensity	0 to +100	17.9	A	15.9	A	18.2	A	17.9	A
	Strawberry flavor intensity	0 to +100	34.3	A	20.4	B	37.5	A	25.2	B
Biochemical measures										
	Glucose	(mg^1^ 100 gFW^−1^)	1903	A	1127	B	2187	A	1807	B
	Fructose	(mg^1^ 100 gFW^−1^)	2048	A	1311	B	2327	A	1973	B
	Sucrose	(mg^1^ 100 gFW^−1^)	1218	A	309	B	1902	A	450	B
	Total sugar	(mg^1^ 100 gFW^−1^)	5169	-	2747	-	6417	-	4229	-
	Relative sucrose	-	0.37	B	0.41	A	0.34	B	0.43	A
	Relative fructose	-	0.40	B	0.48	A	0.36	B	0.47	A
	Relative sucrose	-	0.24	A	0.11	B	0.30	A	0.11	B
	Total volatiles	(ng^1^ gFW^−1^ h^−1^)	19097	A	11543	B	16843	A	16001	A

Comparison of means for temperature (mean of 7 days prior to harvest), consumer ratings, and biochemical measures between early and late season strawberry fruit cultivar ‘Festival’ from season 1 and season 2. Mean comparison accomplished in JMP 8 using Tukey’s HSD. Mean marked A is significantly greater than mean marked B (α = 0.05).

**Figure 3:**
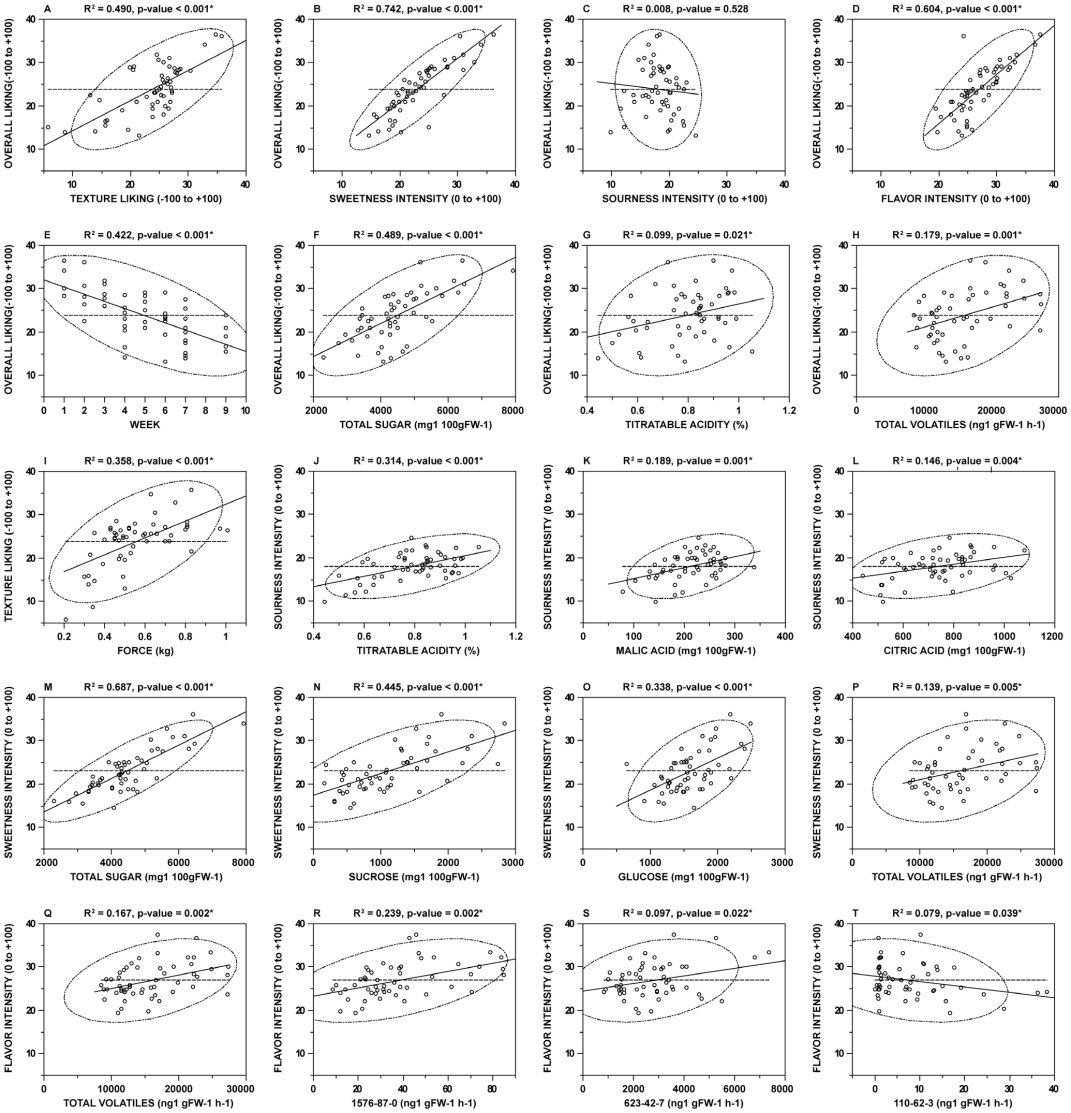
Regression of hedonic and sensory measures to physical and chemical fruit attributes. Hedonic overall liking is regressed against hedonic texture liking (A), sweetness intensity (B), sourness intensity (C), and strawberry flavor intensity (D). Overall liking is fitted to harvest week (E), total sugars (F), titratable acidity (G), and total volatiles (H). Texture liking is examined against puncture force (I). Intensity of sourness is fitted to titratable acidity (J), malic acid (K), and citric acid (L). Sweetness intensity is regressed against total sugars (M), sucrose (N), glucose (O), and total volatiles (P). Strawberry flavor intensity is regressed by total volatiles (Q) and select single volatile compounds 1576-87-0 (R), 623-42-7 (S), and 110-62-3 (T). Coefficient of determination (R^2^) and *p*-value of fit is listed above individual scatterplots and is calculated using bivariate fit in JMP 8. Dashed line represents mean of independent variable, solid line represents linear fit, dashed/dotted ellipse indicates 95% confidence range of data, and asterisk denotes significant fit (α = 0.05).

Solar radiation, minimum temperature and maximum temperature increase gradually within the limits of similar ranges in season 1 and season 2 (Fig. S1 A-D). Relative humidity remains constant during and across seasons (Fig. S1 E, F). Slightly more rain fell in early season 1 than season 2 (Fig. S1 G-H) One manifestation of these environmental changes over a harvest season is the negative relationship between total sugar and harvest week (Table 1). The content of all individual sugars measured decreases between early and late season ‘Festival’ samples; however there is a significant decrease in the proportion of sucrose to total sugar (Table 1). The disproportionate decrease is observed for the collection of samples as well (Fig. S2A-C) (Table S3). Also, a significant correlation is observed across all 54 samples among total volatiles and sucrose (R^2^ = 0.305*) (Fig. S2E) but not glucose (R^2^ = 0.005) (data not shown) or fructose (R^2^ = 0.001) (Fig. S2F). A harvest week associated decrease in total sugars, predominantly sucrose, results in a decrease in volatile content, which ultimately undermines late season overall liking (R^2^ = 0.422*) (Fig. 3E) through sweetness and strawberry flavor intensity.

### Overall Liking is Subject to Ratings of Sweetness, Flavor, and Texture but not Sourness

In order to elucidate factors contributing to a positive strawberry experience, overall liking of strawberry samples is fit against the hedonic measure of texture liking and the sensory intensities of sweetness, sourness, and strawberry flavor intensity (Fig. 3A-D). High correlation with significant fit exists for texture liking (R^2^ = 0.490*) (Fig. 3A), sweetness intensity (R^2^ = 0.742*) (Fig. 3B), and strawberry flavor intensity (R^2^ = 0.604*) (Fig. 3D). However, sourness intensity shows no correlation to overall liking (R^2^ = 0.008) (Fig. 3C). Increasing firmness contributes to greater texture liking (R^2^ = 0.358*) (Fig. 3I), and texture liking has a significant influence on overall liking. Sweetness intensity is the strongest driver of overall liking measured in this study. The correlation between total sugar and overall liking (R^2^ = 0.488*) (Fig. 3F) demonstrates the aggregate sugar metabolites effect on hedonic response to strawberry fruit. Total sugar concentration accounts for nearly half of the observed overall liking variation but is far from a complete measure. Sourness intensity appears to have no influence on the hedonic response to strawberry fruit, but fit of TA to overall liking is significant, even if minor (R^2^ = 0.099*) (Fig. 3G). Total volatiles is the second aggregate metabolite measure having a significant enhancing effect on the overall liking of strawberry (R^2^ = 0.179*) (Fig. 3H). This is not surprising, as strawberry flavor intensity exhibits the second highest correlation to overall liking (Fig. 3D).

### Texture Liking Correlates to Fruit Firmness

The upper limit for hedonics of texture is comparable to that of overall liking and is observed in ‘Festival’ (sn 1, wk 2) with an average of 35.7, however, the low texture liking value of 5.8 for ‘Mara Des Bois’ (sn 1, wk 7) indicates a more drastic disliking of “off” textures than the overall liking of even the lowest rating fruit (Table S2). Firmness of samples is assayed by measuring the force required for a set penetration of the fruit, acting as a proxy for texture. The firmness of the fresh strawberry exhibited nearly a five-fold difference in force, 0.2 kg for ‘Mara des Bois’ (sn 1, wk 7) and 1.0 kg for ‘Festival’ (sn 1, wk 5) (Table S2). Increasing force of penetration, i.e. increasing firmness of berries, is positively correlated with texture liking, indicating a hedonic response to firmer fruit (Fig. 3I). However, the texture liking is less than the expected rating for the two samples with greatest firmness (Fig. 3I).

### Sweetness Intensity is a Result of Sugar Content

Perceived sweetness intensity is the greatest predictor of overall liking. In fact, the same samples scoring the highest and lowest for overall liking, ‘Festival’ (sn 2, wk 1) and ‘Red Merlin’ (sn 1, wk 6), elicit the greatest (36.2) and least (14.59) intense sensations of sweetness (Table S2). The early and late harvest week samples support the observable decline in perceived sweetness intensity across harvest weeks, which is also observable for multiple sugar measures (Fig. S2A-C) (Table 1).

In the 54 samples assayed, the total sugar concentration ranged from 2.29 – 7.93%, a 3.5-fold difference (Table S2). Glucose and fructose concentrations exhibit highly similar ranges to each other, 0.66 – 2.48% and 0.75 – 2.61%, respectively (Table S2), and near-perfect correlation (R^2^ = 0.984*) (data not shown) within a sample. However, the concentration of glucose or fructose is not predictive of sucrose concentration (R^2^ = 0.011 and 0.004, respectively) (data not shown). Sucrose demonstrated a more dynamic state as its concentration dips as low as 0.16% and up to 2.84%, nearly a seventeen-fold difference among all samples.

Sucrose is the single metabolite with the most significant contribution to overall liking (R^2^ = 0.442*) (Table S4). Individually, sucrose (R^2^ = 0.445*) (Fig. 3N), glucose (R^2^ = 0.337*) (Fig. 3O), and fructose (R^2^ = 0.300*) (Table S4) all significantly influence the variation in sweetness intensity. However, total sugar actually only accounts for slightly more than two-thirds of sweetness intensity variation (R^2^ = 0.687*) (Fig. 3M) likely a result of covariation of glucose and fructose. Interestingly, the total volatile content of a sample correlates positively with sweetness intensity, potentially accounting for up to 13.9%* of variation in sweetness intensity (Fig. 3P).

### Sourness Intensity is Partially Explained by Titratable Acidity

Cultivar ‘Red Merlin’ (sn 1, wk 6) elicited the most intense sourness response at 24.6 (Table S2). This same sample rates as the lowest in terms of overall liking and sweetness. Acidity of strawberry fruit is assayed using measures of pH, TA, citric acid and malic acid. The pH of strawberry samples ranges from 3.35 to 4.12, while TA ranges from 0.44% to 1.05%. The range of malic acid across samples is 0.078% to 0.338% while citric acid ranged from 0.441% to 1.080% (Table S2). TA has the greatest correlation to sourness intensity (R^2^ = 0.314*) (Fig. 3J), when compared to pH (R^2^ = 0.118*), malic acid (R^2^ = 0.189*) (Fig. 3K), or citric acid (R^2^ = 0.146*) (Fig. 3L) concentration. Citric acid concentration is approximately three-fold greater than malic acid and has a significant effect on TA (R^2^ = 0.49*) (data not shown). There is no correlation of malic acid to TA (R^2^ = 0.01) (data not shown). The lack of relationship among sourness intensity and overall liking (Fig. 3C) is shadowed by the strong correlations of sweetness intensity (Fig. 3B) and flavor intensity (Fig. 3D) to overall liking. Deficiencies in perceived sweetness and flavor intensity as observed in ‘Red Merlin’ can result in a fruit that is negatively perceived as intensely sour.

### Flavor Intensity Is Influenced by Total and Specific Volatile Content

In this study, strawberry flavor intensity accounts for the retronasal olfaction component of chemical senses, which compliments sourness and sweetness intensities’ contribution to taste. The overall highest sensory intensity is 37.5 (Table S2) for strawberry flavor of ‘Festival’ (sn 2, wk 1), which also rates highest for overall liking and sweetness intensity. Opposite this, FL- 05-85 (sn 1, wk 6) delivers the least intense strawberry flavor experience with a score of 19.4 (Table S2). Total volatiles in ‘Festival’ (sn 2, wk 1) is over 50% greater than in FL 05-85 and seven more volatiles compounds are detected (Table S2). Total volatiles within a sample contribute to strawberry flavor intensity (R^2^ = 0.167*) (Fig. 3Q), but it is not simply the sum of volatile constituents that explain the effect. For instance, the maximum total volatile content detected within a sample, 27.3 µg^1^ gFW^−1^ hr^−1^ from ‘Camarosa’ (sn 1, wk 2), does not result in the greatest flavor intensity (30.5) and the minimum, 8.5 µg^1^ gFW^−1^ hr^−1^ from ‘Sweet Anne’ (sn 2, wk 9), does not rate as the least flavorful (25.8) (Table S2).

The chemical diversity of the resources analyzed allows for the identification of 81 volatile compounds from fresh strawberry fruit (Fig. S3). The majority of compounds are lipid related esters, while lipid related aldehydes account for the majority of volatile mass. Terpenes, furans, and ketones are also represented in the headspace of strawberry. Forty-three of the 81 volatile compounds are not detected (<0.06 ng^1^ gFW^−1^ hr^−1^) in at least one sample. Therefore, 38 volatiles are measured in all samples; appearing to be constant in the genetic resources analyzed (Table S2). No cultivar has detectable amounts of all 81 volatiles. Samples of ‘Festival’, ‘Camino Real’, PROPRIETARY 6, and FL 06-38 are the most volatile diverse, but are lacking detectable amounts benzoic acid, 2-amino-, methyl ester (134-20-3) [5]. This methyl ester of anthranilic acid is detectable in only ‘Mara des Bois’ and ‘Charlotte’ from the final harvest (wk 7) of season 1 (Table S2). ‘Chandler’ (sn 2, wk 4) and ‘Red Merlin’ (sn 1, wk 6) are the least volatile diverse samples lacking detectable amounts of 19 and 17 compounds, respectively (Table S2).

The most abundant ester, butanoic acid, methyl ester (623-42-7)is measured at over 7 µg^1^ gFW^−1^ hr^−1^ from PROPRIETARY 2 (sn 1, wk 3) and has a significant correlation to flavor (R^2^ = 0.097*) (Fig. 3S). A terpene alcohol, 1,6,10-Dodecatrien-3-ol, 3,7,11-trimethyl-, (6E)- (40716-66-3) (nerolidol), with maximum content of over 600 ng^1^ gFW^−1^ hr^−1^ in ‘Sweet Charlie’ is not detected in ‘Red Merlin’. The nerolidol rich ‘Sweet Charlie’ garners greater flavor intensity at 32.2 than deficient ‘Red Merlin’ at 23.95. The impact on flavor intensity by nerolidol (R^2^ = 0.112*) (Table S4) is greater than butanoic acid, methyl ester despite having maximum contents lower by one order of magnitude. Hexanal (66-25-1) is the second most abundant individual compound, an aldehyde detected in all samples, exceeds 11 µg^1^ gFW^−1^ hr^−1^(Table S2), and does not have a significant correlation to flavor intensity (R^2^ = 0.016) (Table S4). Hexanoic acid, ethyl ester (123-66-0) exhibits over 200-fold difference across samples, and also has no bearing on sensory perception (Table S4). Conversely, two minor level aldehydes demonstrate a disparity in effect: 2-pentenal, (2E)- (1576-87-0) is enhancing toward flavor intensity (R^2^ = 0.239*) (Fig. 3R), while pentanal (110-62-3) is the only compound that negatively correlates to flavor (R^2^ = 0.079*) (Fig. 3T). The significant contribution of the 1,6-octadien-3-ol, 3,7-dimethyl- (78-70-6) (linalool) to flavor intensity positively correlates with increasing content (R2 = 0.074*) (Table S4). In ‘Chandler’ 3(2H)-furanone, 4-methoxy-2,5-dimethyl- (4077-47-8) is not detectable, and only has maximum content of 40 ng^1^ gFW^−1^ hr^−1^ in ‘Treasure’ (sn 1 wk 3). The level of this characteristic strawberry furan is significantly impactful on perceived flavor intensity (R^2^ = 0.108*) (Table S4). In total, thirty volatile compounds diverse in structure have a positive relationship to flavor intensity and their significance cannot be derived from content alone.

### Specific Volatiles Enhance Sweetness Intensity Independent of Sugars

Multiple regression of individual volatile compounds against perceived intensity of sweetness is performed independent of glucose, fructose, or sucrose concentration (Table S5). Twenty four volatile compounds show significant correlations (α = 0.05) to perceived sweetness intensity independent of glucose or fructose concentration, twenty-two of which are mutual between the two monosaccharides. Twenty volatiles are found to enhance sweetness intensity independent of sucrose concentration; only six of these volatiles are shared with those independent of glucose and fructose: 1-penten-3-one (1629-58-9); 2(3*H*)-furanone, dihydro-5-octyl- (2305-05-7) (γ-dodecalactone); butanoic acid, pentyl ester (540-18-1); butanoic acid, hexyl ester (2639-63-6); acetic acid, hexyl ester (142-92-7); and butanoic acid, 1-methylbutyl ester. Only three compounds are found to be negatively related to sweetness independent of at least one of the sugars: octanoic acid, ethyl ester (106-32-1) exclusively independent of glucose; 2-pentanone, 4-methyl- (108-10-1) mutually independent of glucose and fructose; and 2-buten-1-ol, 3-methyl-, 1-acetate (1191-16-8) exclusively independent of sucrose.

## Discussion

Exploitation of genetic diversity and environmental variation allows for a wide range of consumer hedonic and sensory responses. The cultivars in this study represent a large proportion of commercial strawberry acreage in North America, advanced breeding selections, and European cultivars. A genetic collection aimed at enhancing the diversity of physical and chemical constituents, as well as consumer experiences. Despite the perennial life cycle of strawberry much commercial production uses annual methods, which in sub-tropical Florida allows for continual harvest of ripe fruit from late November through March. A nearly three-fold difference in overall liking of strawberry is observable within all samples. The highest and lowest rating samples are ‘Festival’ of the first week in the second season and ‘Red Merlin’ of the sixth week in the first season. These two cultivars are the product of separate breeding programs, have distinct genetic backgrounds, and therefore distinct biochemical inventories. Harvested at opposite ends of the seasons the early and late season fruit are subjected to different environmental conditions, further attenuating genetic differences. The diversity of strawberries samples assayed and range of consumer liking captured (Fig. 2) indicates the chemical diversity of strawberry cultivars is not only perceivably different but certain profiles are more highly preferable.

Elevated texture liking, sweetness intensity, and strawberry flavor intensity significantly increases overall liking, while sourness intensity alone has no detectable relationship to overall liking (Fig. 3A-D). Integration and synthesis of response to sensory signals of taste, olfaction, and tactile sensation constitute an eating experience [9] and drive overall liking. The senses of taste and olfaction sample the chemicals present in food *e.g.* sugars, acids, and volatile chemical compounds. These elicitors attenuate the perception and hedonics of food [38], [39]. Ratings of strawberry fruit are correlated to specific chemical or physical attributes, especially sweetness (Fig. 3B) and flavor intensity (Fig. 3D), the two greatest drivers of overall liking.

Much work has been done to measure sugars and volatile compounds in strawberry fruit in an attempt to understand sweetness and flavor, and these aims are in line with consumer demand. A consumer survey using 36 attributes of strawberry determined “sweetness” and “complex flavor” as consistent favorable characteristics of the ideal strawberry experience [14]. Previous work in tomato [34] and this current study on strawberry surveyed participants for ideal ratings of the respective fruits. Using the same gLMS scales employed in the current study, scores for ideal strawberry and tomato overall liking, sourness intensity, and flavor intensity are highly similar. Ideal flavor evoked the highest mean response of 45 for both, exemplifying its importance to the consumer. Interestingly, a large disparity for ideal sweetness intensity is found; 42 and 33 for strawberry and tomato, respectively. Ideal sweetness intensity is much greater in strawberry, potentially due to differences in consumption. Strawberry is often consumed fresh and is a delicacy or dessert fruit, while tomato is savory and often an ingredient in complex recipes. Therefore, the desire for sweetness may be greater in strawberry.

The overall liking of strawberry fruit is significantly related to texture liking (Fig. 3A), and increasing fruit firmness accounts for more than a third of increasing texture liking (Fig. 3I). The five-fold variation in firmness can be attributed to variation in fruit development or softening (Table S2). Strawberry fruit development consists of division, expansion, and ripening [7]. Developmentally regulated, ripening associated fruit softening is multifaceted [19], including catalytic cell wall disassembly [20] and dissolution of cell-to-cell adhesion [3]. The relationship between texture liking and firmness does not appear entirely linear, because the two firmest samples are close to average texture liking (Fig. 3I). Excessively firm fruits may be perceived as under ripe while those with less firmness may be considered over ripe; affecting texture liking. Fruit can progress through ripening, from under to over ripe, in ten days [7], exemplifying the narrow window in which multiple facets of fruit quality must synchronize.

Despite a moderate range of intensity, perceived sourness has little to no bearing on overall liking (Fig. 3C). Just over 30% of sourness intensity variation can be accounted for by positive correlation with TA. The concentrations of citric acid and malic acid metabolites are likely additive toward the effect of TA on sourness intensity, and in fact both organic acids have significant correlations to sourness intensity (Fig. 3K-L). Despite a lack of influence by sourness intensity on overall liking, metabolites of sourness have a critical role in fruit biochemistry. Increased TA shows a significant minor correlation with overall liking (Table S4) and correlates significantly with total sugar (data not shown). This co-linearity may be due to accumulation of sugars and subsequent biosynthesis of organic acids during ripening of fruit [7], [8], [16]. Citric acid is the predominant organic acid in ripe fruit [40] and its concentration is fairly stable during ripening. Also, it is known to act as an intermediate between imported sucrose and fatty acid biosynthesis [16], which may facilitate enhancement of overall liking through volatile biosynthesis.

The consumer rating of sweetness intensity is the primary factor contributing to overall liking, and sweetness is the component of taste perception facilitating the detection of sugars. Sugars are simple carbohydrates, a readily available form of energy, and the degree of correlation among sweetness and overall liking is due to hedonic effect [38]. Variation in sweetness intensity is best explained by sugar content (Fig. 3L). Previously, soluble solid content (SSC) has been used as a valid indicator of sweetness in strawberry [1], [28]. However this is an aggregate measure, as previous quantification of individual sugars within a strawberry identifies sucrose, glucose, and fructose as the predominant soluble solids [1], [8], [15], [40]. Sucrose concentrations observed across samples is responsible for more variation in total sugar, sweetness intensity and overall liking than any other individual compound (Table S4). Metabolites contributing to perceived sweetness intensity have the greatest influence on the overall hedonics of strawberry. A significant decrease in sweetness intensity occurs between early and late season fruit, and unfortunately overall liking decreases as well (Table 1) (Fig. 3E).

Drastic fruit quality differences between early and late season fruit result in lower consumer response (Table 1) (Fig. 3E), which is likely due to environmental changes (Fig. S1) or plant maturity. A significant difference in the mean temperature one week prior to harvest is likely a causative factor (Table 1). Monitored development of ‘Festival’ fruit under elevated temperature decreases the fruit development period from 36 days at 15°C to 24 days at 22°C. Also, a simultaneous decrease in SSC is observed, both independent of flowering date i.e. plant maturity [26], [27]. The mean temperature of the week prior to harvest for early and late season ‘Festival’ fruit are 15°C and 20°C for the first season and 14°C and 19°C for the second season (Table 1). These differences in environment likely alter whole plant physiology and more specifically fruit biochemistry during development and ripening, affecting fruit quality. During strawberry fruit development sucrose is continually translocated from photosynthetic tissue, while a consistently high sucrose invertase activity in fruit hydrolyzes sucrose into glucose and fructose, maintaining sink strength of fruit [15] and in turn feed biosynthetic pathways [16]. Total and individual sugars decrease in ripe fruit during both seasons as the plant is subjected to increasing temperatures (Table 1). Increased maturation rate hastens fruit development, potentially decreasing cumulative period sucrose is imported to fruit, and inhibiting sucrose accumulation to affect other fruit quality attributes. These factors are likely causative of the observable decrease in sweetness and flavor intensity as the season progresses.

Although total sugar decreases between early and late fruit, a disproportionate amount of the decrease is attributed to sucrose (Table 1), which indicates sucrose as the waning constituent of sugar content (Fig. S2A-C). Glucose and fructose concentrations are tightly correlated to each other, show less seasonal influence than sucrose, and lack correlation to sucrose. These observations are indicative of tighter biochemical regulation of glucose and fructose than sucrose, which has the greatest variability in concentration among the three sugars. Total volatile content has an indirect dependence on sucrose concentration (Fig. S2E), and a decrease in total volatiles is observed between early and late season strawberry (Table 1). Influence of harvest date on headspace of fresh strawberry fruit is known [41], [42]. Increased volatile content is likely dependent on more free sucrose, *i.e.* a larger imported reserve, facilitating greater flux through primary and secondary metabolism. Generation of glucose and fructose initiates a complex network of primary and secondary metabolism specific to ripening strawberry fruit, in which sucrose is principal and limiting to the strawberry fruit biosynthetic pathways [16]. Upregulation of biosynthetic genes associated with volatile secondary metabolites [43] and the consumption of primary metabolite classes of fatty acids and amino acids, precursors of volatile compounds, happens in the final stages of ripening [16]. This sucrose dependent metabolic shift culminates in peak volatile content and diversity [8].

Strawberry flavor intensity is the second greatest determinant of overall liking (Fig. 3D) and accounts for perception of volatile compounds through retronasal olfaction. A significant positive relationship exists among total volatile content and the flavor intensity for a given sample, however, total volatile content is not entirely explanatory of flavor intensity. The maximum rating for strawberry flavor intensity by ‘Festival’ (sn 2, wk1) is the greatest consumer response evoked within this study (Table S2), highlighting the significance of sensory perception of aroma. However, this sample only has slightly more than 60% of total volatile mass of the greatest sample. The extent of volatile phenotype diversity is great enough across strawberry fruit to not only be discerned but be preferred.

Within the genetic resources of *Fragaria x ananassa* analyzed in this study 81 compounds are reproducibly detected, but not one cultivar has detectable amounts of all compounds. The amount of individual volatile compounds within fruit can have a significant influence on flavor intensity, but which volatiles are determinant of flavor has a lack of consensus. Previous determination of flavor relevance relied on approaches in which importance of volatiles is at least initially based on abundance. Determination of flavor descriptors or thresholds of isolated compounds were determined using human perception via orthonasal olfaction [4], [5], [23]–[25], negating the complex system of strawberry fruit or actual flavor relevant retronasal olfaction.

Of the forty-six volatile compounds cited as relevant to strawberry flavor in five studies [4], [5], [23]–[25] only seven are mutual to at least three of the studies, exemplifying the lack of agreement in defining flavor-relevant constituents. This consensus includes butanoic acid, methyl ester; butanoic acid, ethyl ester; hexanoic acid, methyl ester (106-70-7); hexanoic acid, ethyl ester; linalool; butanoic acid, 2-methyl- (116-53-0); and 3(2*H*)-furanone, 4-methoxy-2,5-dimethyl-, all of which are quantified in this report. These compounds exhibit adequate variability in fruit samples to discern dose dependent effect on flavor intensity. However, only linalool; butanoic acid, ethyl ester; butanoic acid, methyl ester; and 3(2*H*)-furanone, 4-methoxy-2,5-dimethyl- show significant positive correlation with flavor intensity (Table S4). These compounds that are found to influence flavor intensity represent diverse classes, terpene alcohol, two esters, and a furan, respectively, while the three compounds not fitting to flavor are all esters. With esters accounting for the majority of chemical compounds detected in strawberry it is possible that too much emphasis is placed on the chemical class for flavor, or that in a complex mixture less are perceivable than when smelled individually.

Over one third of volatiles in this study significantly correlate with strawberry flavor intensity, potentially enhancing perception of a complex and highly variable volatile mixture (Table S4), seventeen of which are not of previous strawberry flavor focus. Two of these unrecognized compounds, 1-hexanol (111-71-7) and butanoic acid, 3-methyl-, butyl ester (109-19-3), are present in the most flavorful strawberry sample but undetected in the least flavorful (Table S2). This pair of compounds as well as pentanoic acid, ethyl ester (539-82-2) and butanoic acid, 3-methyl-, octyl ester (7786-58-5), also present/absent in the most/least flavorful, have relatively minor amounts but show evidence of enhancing perceived sweetness intensity independent of individual sugars. Relatively low abundance volatiles are indicated as new impactful components of strawberry flavor.

Thirty-eight volatile compounds are observed to significantly enhance the perceived intensity of sweetness; twenty-two mutually independent of glucose and fructose, fourteen uniquely independent of sucrose, and six compounds mutually independent of all three sugar: 1-penten-3-one; 2(3*H*)-furanone, dihydro-5-octyl- (γ-dodecalactone); butanoic acid, pentyl ester; butanoic acid, hexyl ester; acetic acid, hexyl ester; and butanoic acid, 1-methylbutyl ester (Table S5). In tomato, similar analysis of a volatile subset identifies three compounds enhancing sweetness intensity independent of fructose: geranial; 1-butanol, 3-methyl- (123-51-3); and butanal, 2-methyl- (96-17-3) [34]. These compounds are not identified in the current study; therefore the effect cannot be confirmed in a second system. Botanically, tomato is considered a true fruit and demonstrates climacteric ripening, while strawberry fruit is non-climacteric and considered an aggregate accessory fruit. The developmental origin of the flesh which is consumed is divergent, exhibiting unique biochemistries, but the observance of volatile compounds potentially enhancing perceived sweetness appears to be widespread in fruit.

Orthonasal olfaction is the result of smelling *i.e.* bringing odor in through the nose, while retronasal olfaction is elicited by odorants traveling from oral cavity or esophagus up to nasal cavity [44]. Orthonasal olfaction introduces volatile compounds to the nasal epithelium via inhalation, while retronasal olfaction is achieved during exhalation [45]. Specifically, the path of odorants distinguishes the manner of interaction between consumer and potential food, with orthonasal contributing to aroma and retronasal to flavor. Integration of sensory stimuli relies on projection of signals to various structures of the brain. Interestingly, portions of orthonasal (smell) and retronasal (flavor) olfaction project to different brain areas for processing [46], while taste activation partly overlaps that of retronasal olfaction for integration to produce flavor [47]. Co-activation of taste and retronasal olfaction, but not orthonasal, is shown to elicit responses at otherwise independently sub-threshold levels, exemplifying the ability of multiple sensory integration to intensify one another [48]. Mechanical blockage of retronasal olfaction during tasting of solutions significantly reduces the ability to correctly identify solute, including sucrose [45]. Combination of taste and retronasal olfaction results in a sensory system more adapt at analyzing the chemical content of food, but cross communication also facilitates manipulation of the system.

The food industry knows of the intensification of volatile sensations by the addition of small amounts of sweeteners to solutions containing volatiles [49]. The ability of volatiles to enhance taste is also a known phenomenon [38]. Enhancement of perceived sweetness is demonstrated by addition of volatiles amyl acetate (banana) [50] and citral [51]. Multiple studies show the ability of strawberry aroma to intensify the sweetness of a sugar solution [52], [53], as well as pineapple, raspberry, passion fruit, lychee, and peach [53], [54]. Also, sweetness enhancement has been achieved with vanilla [55], caramel [53], [56], and chocolate [45] indicating this phenomenon is not only associated with fruit volatiles. Studies to determine perceptional differences when tomato is spiked with sugars, acids, and volatiles indicates cross talk between taste and olfaction, in which volatiles impact perception of sweetness and vice versa [57]. Individual volatile compounds have been implicated in tomato to intensify perceived sweetness independent of sugar content [34], [58]. The results here narrow the previous effect of enhanced sweetness by strawberry aroma, a variable mixture, to individual compounds in the fruit. These volatiles are not present at the highest amounts in fruits and most have not been targets of flavor analysis. Also, most appear to be associated with lipid metabolism, like many other volatiles quantified in this work, yet their presence or increased content has an enhancing effect on perceived sweetness independent of sugars. Technically, sweetness is a facet of taste [38]. Therefore a means to convey sweetness via aroma can serve as an attractant to seed dispersers of wild strawberry, or perhaps it is a result of artificial selection [59] to enhance a limited sugar capacity in commercial fruit.

## Conclusions

Strawberry fruit ripening culminates as the flesh softens, volatile emission peaks, and sugars accumulate. This highly coordinated process results in fruit with strong liking due primarily to texture, flavor, and sweetness. However, cultivar, environmental conditions, and their interactions influence fruit attributes, altering the composition of strawberry. This diversity allows for a spectrum of experiences such that the hedonics and intensities of these sensations can vary greatly. The importance of sucrose to sweetness intensity is evident, and the correlation of total volatiles to sucrose highlights the dependence of secondary metabolism to primary metabolism. Individual volatiles correlate to strawberry flavor intensity, helping to better define distinct, perceptually impactful compounds from the larger mixture of the fruit. The dependence of liking on sweetness and strawberry flavor is undermined by environmental pressures that reduce sucrose and total volatile content. A cultivar that exhibits minimal seasonal environmental influence presents itself as a breeding ideotype, as maintenance of sucrose concentration may alleviate loss of overall liking. Selection for increased amounts of volatile compounds that act independently of sugars to enhance sweetness can serve as an alternate approach. The volatiles described herein are sampled mainly from current commercial cultivars and are therefore feasible targets for varietal improvement in the short-term, whereas future studies will be necessary to identify sweet-enhancing volatiles not already present in elite germplasm.

## Supporting Information

Figure S1 **Season Environmental Conditions.** Daily maximum and minimum temperatures (A and B), daily average solar radiation (C and D), daily average relative humidity (E and F), and daily total rain fall (G and H) during the 2011 (A, C, E, and G) and 2012 (B, D, F, and H) seasons. Data for Balm, FL obtained from Florida Automated Weather Network (http://fawn.ifas.ufl.edu/data/reports). Data spans three weeks prior to first harvest through last harvest of each season with individual harvests indicated by dotted vertical line and harvest week number. Dashed horizontal lines represent means of environmental measures. Solid lines are the bivariate fit of environmental measure across season. Coefficients of determination (R^2^) and *p*-value of fit is listed above individual scatterplots and are calculated using bivariate fit in JMP 8.
(TIF)

Figure S2 **Individual sugars and total volatiles regressed against season progression.** Regression of sucrose (A), glucose (B), fructose (C), and total volatiles (D) by harvest week during the seasons. Total volatile content is regressed against sucrose (E) and fructose (F). Sucrose (A) and total volatiles (D) demonstrate a significant negative fit to harvest week, unlike glucose (B) and fructose (C). A strong relationship between total volatile emmission and sucrose concentration is found (E) that is not observed between total volatiles and glucose (data not shown) and fructose (F). Coefficient of determination (R^2^) and *p*-value of fit is listed above individual scatterplots and is calculated using bivariate fit in JMP 8. Dashed line represents mean of independent variable, solid line represents linear fit, dashed/dotted ellipse indicates 95% confidence range of data, and asterisk denotes significant fit (α = 0.05).
(TIF)

Figure S3 **Chemical structure of volatile compounds.** Chemical structure of volatile compounds quantified in strawberry. Sorted by increasing retention time (left to right, top row to bottom row), identified by CAS Registry Number.
(TIF)

Table S1 **CAS registry number, chemical name, and formula index.** Chemical Abstract Services (CAS) registry numbers were used to query SciFinder® substances database for associated chemical name and molecular formula.
(DOCX)

Table S2 **Full data table.** Means and standard errors of replicates for all measures for each sample assayed. Includes consumer panel measures, internal and external color, puncture force, organic acids, sugars, SSC, pH, TA, and volatile compounds. High and low value, median, and fold difference for each column displayed above means table.
(XLSX)

Table S3 **Fruit attributes bivariate fit during season.** Regression of harvest week during season (X) on panel responses and metabolite concentration (Y). Coefficient of determination (R^2^), correlation coefficient, p-value, sample size (n), mean and standard deviation of X and Y derived from bivariate fit in JMP 8.
(DOCX)

Table S4 **Fruit quality bivariate fit.** Regression of chemical and physical measures of fruit (X) to panel responses (Y). Coefficient of determination (R^2^), correlation coefficient, p-value, sample size (n), mean and standard deviation of X and Y derived from bivariate fit in JMP 8.
(DOCX)

Table S5 **Multiple regression for identification of sweetness enhancing volatiles.** Individual volatile compound concentrations are regressed against perceived sweetness intensity independent of effect from glucose, fructose, or sucrose, separately. Thirty compounds (*) (α = 0.05) were found to enhance intensity of sweetness independent of at least one of the three sugars. Six compounds (bold) were found to significantly enhance intensity of sweetness independent of all three sugars.
(DOCX)
