# Supplementary figures and images for: Strawberry Flavor: Diverse Chemical Compositions, a Seasonal Influence, and Effects on Sensory Perception
Source: PLoS One. 2014 Feb 11;9(2):e88446. doi: 10.1371/journal.pone.0088446 (PMC3921181; doi:10.1371/journal.pone.0088446)

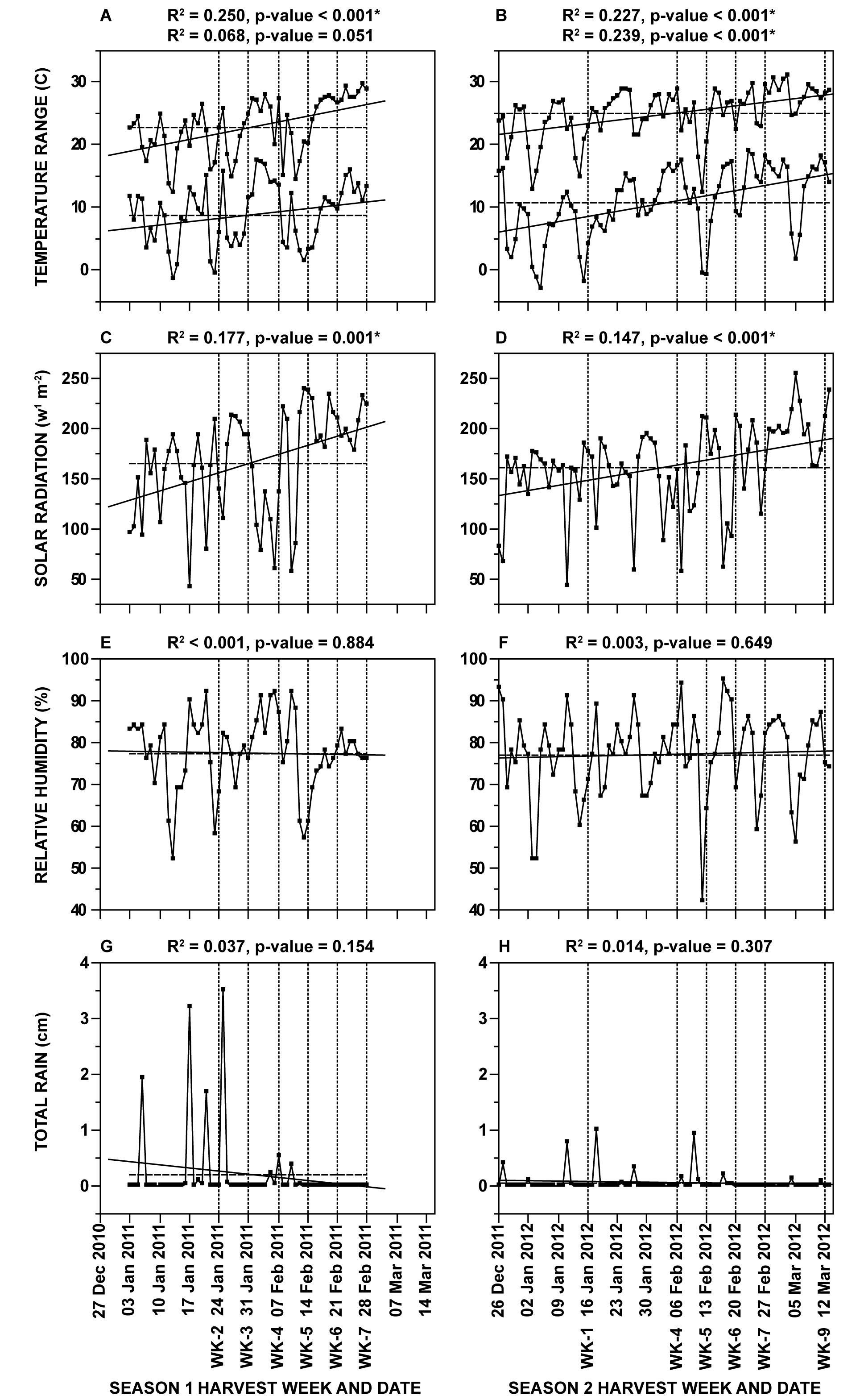

Supplement: Figure S1 — Season Environmental Conditions. Daily maximum and minimum temperatures (A and B), daily average solar radiation (C and D), daily average relative humidity (E and F), and daily total rain fall (G and H) during the 2011 (A, C, E, and G) and 2012 (B, D, F, and H) seasons. Data for Balm, FL obtained from Florida Automated Weather Network (http://fawn.ifas.ufl.edu/data/reports). Data spans three weeks prior to first harvest through last harvest of each season with individual harvests indicated by dotted vertical line and harvest week number. Dashed horizontal lines represent means of environmental measures. Solid lines are the bivariate fit of environmental measure across season. Coefficients of determination (R2) and p-value of fit is listed above individual scatterplots and are calculated using bivariate fit in JMP 8. (TIF) [file pone.0088446.s001.tif]

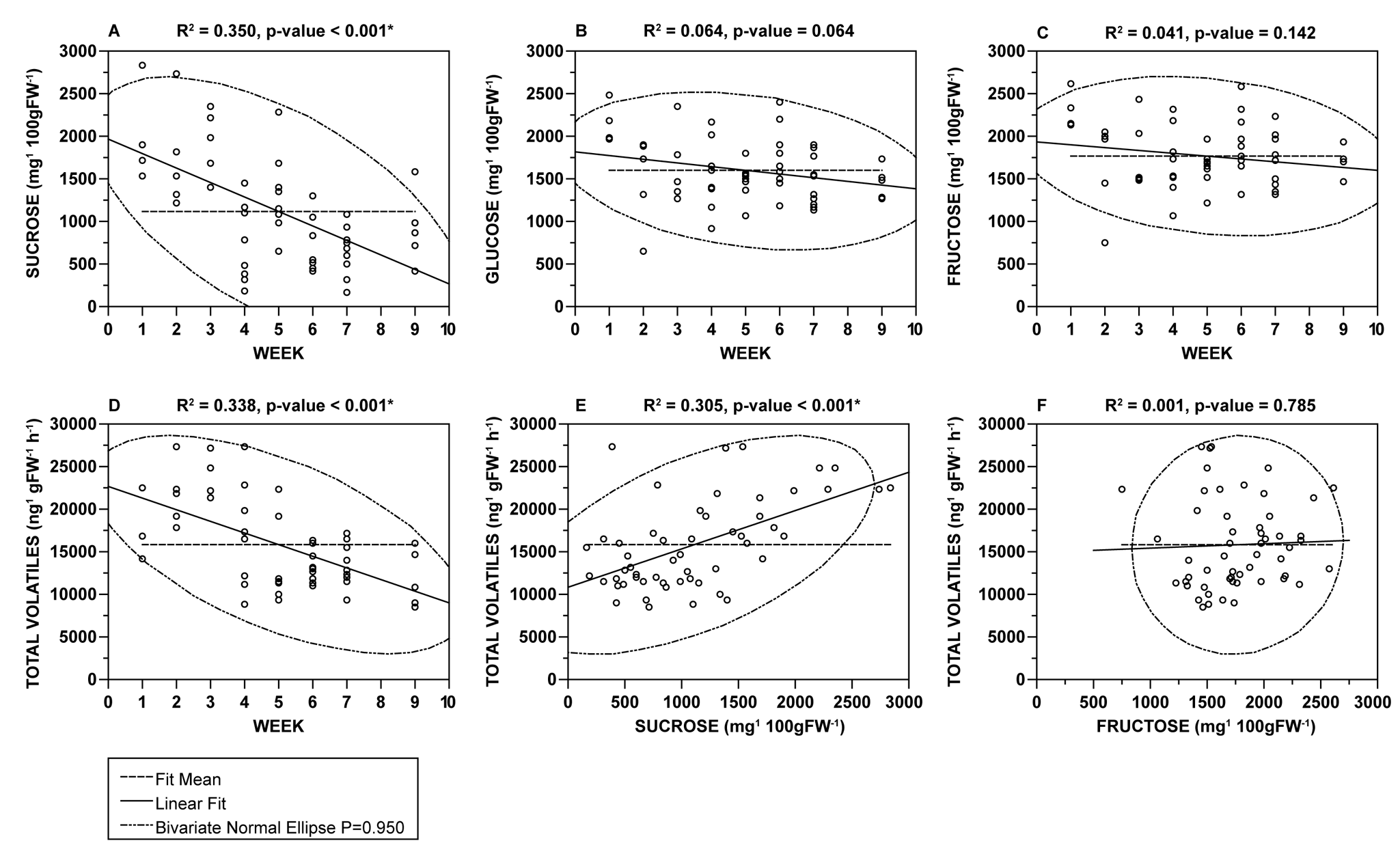

Supplement: Figure S2 — Individual sugars and total volatiles regressed against season progression. Regression of sucrose (A), glucose (B), fructose (C), and total volatiles (D) by harvest week during the seasons. Total volatile content is regressed against sucrose (E) and fructose (F). Sucrose (A) and total volatiles (D) demonstrate a significant negative fit to harvest week, unlike glucose (B) and fructose (C). A strong relationship between total volatile emmission and sucrose concentration is found (E) that is not observed between total volatiles and glucose (data not shown) and fructose (F). Coefficient of determination (R2) and p-value of fit is listed above individual scatterplots and is calculated using bivariate fit in JMP 8. Dashed line represents mean of independent variable, solid line represents linear fit, dashed/dotted ellipse indicates 95% confidence range of data, and asterisk denotes significant fit (α = 0.05). (TIF) [file pone.0088446.s002.tif]

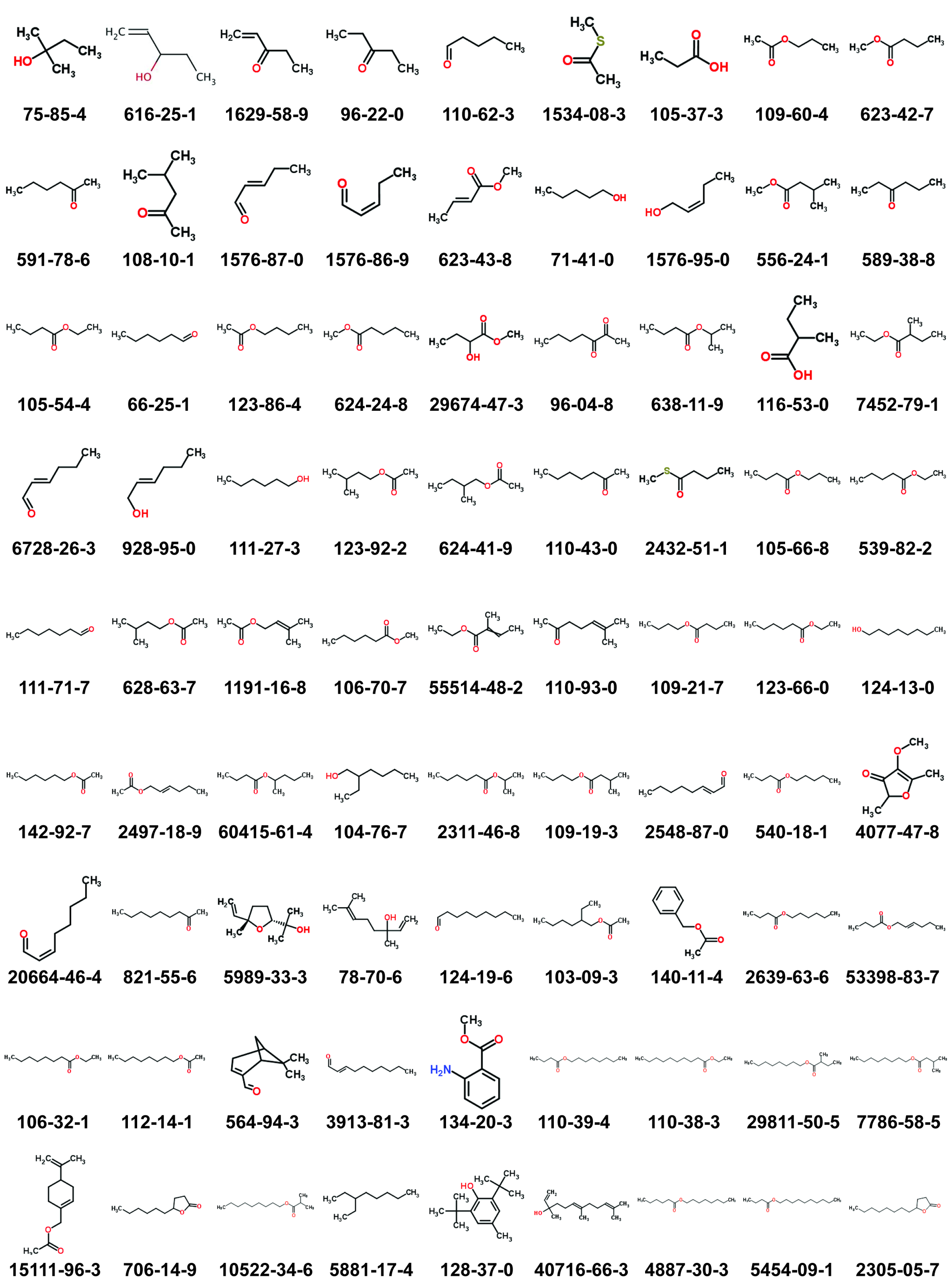

Supplement: Figure S3 — Chemical structure of volatile compounds. Chemical structure of volatile compounds quantified in strawberry. Sorted by increasing retention time (left to right, top row to bottom row), identified by CAS Registry Number. (TIF) [file pone.0088446.s003.tif]
