# Supplementary material for: Strawberry Flavor: Diverse Chemical Compositions, a Seasonal Influence, and Effects on Sensory Perception
Source: PLoS One. 2014 Feb 11;9(2):e88446. doi: 10.1371/journal.pone.0088446 (PMC3921181; doi:10.1371/journal.pone.0088446)
Supplement: Table S1 — CAS registry number, chemical name, and formula index. Chemical Abstract Services (CAS) registry numbers were used to query SciFinder® substances database for associated chemical name and molecular formula. (DOCX) [file pone.0088446.s004.docx]

**Table S1. CAS registry number, chemical name, and formula index.**

| **CAS Registry Number** | **Chemical Name** | **Formula** |
| --- | --- | --- |
| 75-85-4 | 2-Butanol, 2-methyl- | C_5_ H_12_ O |
| 616-25-1 | 1-Penten-3-ol | C_5_ H_10_ O |
| 1629-58-9 | 1-Penten-3-one | C_5_ H_8_ O |
| 96-22-0 | 3-Pentanone | C_5_ H_10_ O |
| 110-62-3 | Pentanal | C_5_ H_10_ O |
| 1534-08-3 | Ethanethioic acid, *S*-methyl ester (9CI) | C_3_ H_6_ O S |
| 105-37-3 | Propanoic acid, ethyl ester | C_5_ H_10_ O_2_ |
| 109-60-4 | Acetic acid, propyl ester | C_5_ H_10_ O_2_ |
| 623-42-7 | Butanoic acid, methyl ester | C_5_ H_10_ O_2_ |
| 591-78-6 | 2-Hexanone | C_6_ H_12_ O |
| 108-10-1 | 2-Pentanone, 4-methyl- | C_6_ H_12_ O |
| 1576-87-0 | 2-Pentenal, (2E)- | C_5_ H_8_ O |
| 1576-86-9 | 2-Pentenal, (2*Z*)- | C_5_ H_8_ O |
| 623-43-8 | 2-Butenoic acid, methyl ester, (2E)- | C_5_ H_8_ O_2_ |
| 71-41-0 | 1-Pentanol | C_5_ H_12_ O |
| 1576-95-0 | 2-Penten-1-ol, (2Z)- | C_5_ H_10_ O |
| 556-24-1 | Butanoic acid, 3-methyl-, methyl ester | C_6_ H_12_ O_2_ |
| 589-38-8 | 3-Hexanone | C_6_ H_12_ O |
| 105-54-4 | Butanoic acid, ethyl ester | C_6_ H_12_ O_2_ |
| 66-25-1 | Hexanal | C_6_ H_12_ O |
| 123-86-4 | Acetic acid, butyl ester | C_6_ H_12_ O_2_ |
| 624-24-8 | Pentanoic acid, methyl ester | C_6_ H_12_ O_2_ |
| 29674-47-3 | Butanoic acid, 2-hydroxy-, methyl ester | C_5_ H_10_ O_3_ |
| 96-04-8 | 2,3-Heptanedione | C_7_ H_12_ O_2_ |
| 638-11-9 | Butanoic acid, 1-methylethyl ester | C_7_ H_14_ O_2_ |
| 116-53-0 | Butanoic acid, 2-methyl- | C_5_ H_10_ O_2_ |
| 7452-79-1 | Butanoic acid, 2-methyl-, ethyl ester | C_7_ H_14_ O_2_ |
| 6728-26-3 | 2-Hexenal, (2E)- | C_6_ H_10_ O |
| 928-95-0 | 2-Hexen-1-ol, (2E)- | C_6_ H_12_ O |
| 111-27-3 | Heptanal | C_7_ H_14_ O |
| 123-92-2 | 1-Butanol, 3-methyl-, 1-acetate | C_7_ H_14_ O_2_ |
| 624-41-9 | 1-Butanol, 2-methyl-, 1-acetate | C_7_ H_14_ O_2_ |
| 110-43-0 | 2-Heptanone | C_7_ H_14_ O |
| 2432-51-1 | Butanethioic acid, S-methyl ester | C_5_ H_10_ O S |
| 105-66-8 | Butanoic acid, propyl ester | C_7_ H_14_ O_2_ |
| 539-82-2 | Pentanoic acid, ethyl ester | C_7_ H_14_ O_2_ |
| 111-71-7 | 1-Hexanol | C_6_ H_14_ O |
| 628-63-7 | Acetic acid, pentyl ester | C_7_ H_14_ O_2_ |
| 1191-16-8 | 2-Buten-1-ol, 3-methyl-, 1-acetate | C_7_ H_12_ O_2_ |
| 106-70-7 | Hexanoic acid, methyl ester | C_7_ H_14_ O_2_ |
| 55514-48-2 | 2-Butenoic acid, 2-methyl-, ethyl ester | C_7_ H_12_ O_2_ |
| 110-93-0 | 5-Hepten-2-one, 6-methyl- | C_8_ H_14_ O |
| 109-21-7 | Butanoic acid, butyl ester | C_8_ H_16_ O_2_ |
| 123-66-0 | Hexanoic acid, ethyl ester | C_8_ H_16_ O_2_ |
| 124-13-0 | Octanal | C_8_ H_16_ O |
| 142-92-7 | Acetic acid, hexyl ester | C_8_ H_16_ O_2_ |
| 2497-18-9 | 2-Hexen-1-ol, 1-acetate, (2E)- | C_8_ H_14_ O_2_ |
| 60415-61-4 | Butanoic acid, 1-methylbutyl ester | C_9_ H_18_ O_2_ |
| 104-76-7 | 1-Hexanol, 2-ethyl- | C_8_ H_18_ O |
| 2311-46-8 | Hexanoic acid, 1-methylethyl ester | C_9_ H_18_ O_2_ |
| 109-19-3 | Butanoic acid, 3-methyl-, butyl ester | C_9_ H_18_ O_2_ |
| 2548-87-0 | 2-Octenal, (2E)- | C_8_ H_14_ O |
| 540-18-1 | Butanoic acid, pentyl ester | C_9_ H_18_ O_2_ |
| 4077-47-8 | 3(2H)-Furanone, 4-methoxy-2,5-dimethyl- | C_7_ H_10_ O_3_ |
| 20664-46-4 | 2-Octenal, (2Z)- | C_8_ H_14_ O |
| 821-55-6 | 2-Nonanone | C_9_ H_18_ O |
| 5989-33-3 | 2-Furanmethanol, 5-ethenyltetrahydro-α,α,5-trimethyl-, (2R,5S)-rel- | C_10_ H_18_ O_2_ |
| 78-70-6 | 1,6-Octadien-3-ol, 3,7-dimethyl- | C_10_ H_18_ O |
| 124-19-6 | Nonanal | C_9_ H_18_ O |
| 103-09-3 | Acetic acid, 2-ethylhexyl ester | C_10_ H_20_ O_2_ |
| 140-11-4 | Acetic acid, phenylmethyl ester | C_9_ H_10_ O_2_ |
| 2639-63-6 | Butanoic acid, hexyl ester | C_10_ H_20_ O_2_ |
| 53398-83-7 | Butanoic acid, (2E)-2-hexen-1-yl ester | C_10_ H_18_ O_2_ |
| 106-32-1 | Octanoic acid, ethyl ester | C_10_ H_20_ O_2_ |
| 112-14-1 | Acetic acid, octyl ester | C_10_ H_20_ O_2_ |
| 564-94-3 | Bicyclo[3.1.1]hept-2-ene-2-carboxaldehyde, 6,6-dimethyl- | C_10_ H_14_ O |
| 3913-81-3 | 2-Decenal, (2E)- | C_10_ H_18_ O |
| 134-20-3 | Benzoic acid, 2-amino-, methyl ester | C_8_ H_9_ N O_2_ |
| 110-39-4 | Butanoic acid, octyl ester | C_12_ H_24_ O_2_ |
| 110-38-3 | Decanoic acid, ethyl ester | C_12_ H_24_ O_2_ |
| 29811-50-5 | Butanoic acid, 2-methyl-, octyl ester | C_13_ H_26_ O_2_ |
| 7786-58-5 | Butanoic acid, 3-methyl-, octyl ester | C_13_ H_26_ O_2_ |
| 15111-96-3 | 1-Cyclohexene-1-methanol, 4-(1-methylethenyl)-, 1-acetate | C_12_ H_18_ O_2_ |
| 706-14-9 | 2(3H)-Furanone, 5-hexyldihydro- | C_10_ H_18_ O_2_ |
| 10522-34-6 | Propanoic acid, 2-methyl-, nonyl ester | C_13_ H_26_ O_2_ |
| 5881-17-4 | Octane, 3-ethyl- | C_10_ H_22_ |
| 128-37-0 | Phenol, 2,6-bis(1,1-dimethylethyl)-4-methyl- | C_15_ H_24_ O |
| 40716-66-3 | 1,6,10-Dodecatrien-3-ol, 3,7,11-trimethyl-, (6E)- | C_15_ H_26_ O |
| 4887-30-3 | Hexanoic acid, octyl ester | C_14_ H_28_ O_2_ |
| 5454-09-1 | Butanoic acid, decyl ester | C_14_ H_28_ O_2_ |
| 2305-05-7 | 2(3H)-Furanone, dihydro-5-octyl- | C_12_ H_22_ O_2_ |

Chemical Abstract Services (CAS) registry numbers were used to query SciFinder® substances database for associated chemical name and molecular formula.
