# Supplementary material for: Strawberry Flavor: Diverse Chemical Compositions, a Seasonal Influence, and Effects on Sensory Perception
Source: PLoS One. 2014 Feb 11;9(2):e88446. doi: 10.1371/journal.pone.0088446 (PMC3921181; doi:10.1371/journal.pone.0088446)
Supplement: Table S3 — Fruit attributes bivariate fit during season. Regression of harvest week during season (X) on panel responses and metabolite concentration (Y). Coefficient of determination (R2), correlation coefficient, p-value, sample size (n), mean and standard deviation of X and Y derived from bivariate fit in JMP 8. (DOCX) [file pone.0088446.s006.docx]

**Table S3. Fruit attributes bivariate fit during season.**

| **X** | **Y** | **R^2^** | **CORR COEFF** | **p-VALUE** | **n** | **MEAN X** | **STD DEV X** | **MEAN Y** | **STD DEV Y** |
| --- | --- | --- | --- | --- | --- | --- | --- | --- | --- |
| WEEK | 1629-58-9 | 0.489 | -0.699 | 0.000 | 54 | 5.0 | 2.2 | 117.9 | 65.9 |
| WEEK | 1576-87-0 | 0.485 | -0.697 | 0.000 | 54 | 5.0 | 2.2 | 37.5 | 20.1 |
| WEEK | SWEETNESS INTENSITY | 0.471 | -0.686 | 0.000 | 54 | 5.0 | 2.2 | 23.0 | 4.8 |
| WEEK | SSC | 0.444 | -0.666 | 0.000 | 54 | 5.0 | 2.2 | 7.4 | 1.4 |
| WEEK | STRAWBERRY FLAVOR INTENSITY | 0.430 | -0.656 | 0.000 | 54 | 5.0 | 2.2 | 26.9 | 3.9 |
| WEEK | OVERALL LIKING | 0.422 | -0.650 | 0.000 | 54 | 5.0 | 2.2 | 23.8 | 5.7 |
| WEEK | 1576-86-9 | 0.402 | -0.634 | 0.000 | 54 | 5.0 | 2.2 | 37.8 | 21.7 |
| WEEK | SUCROSE | 0.350 | -0.592 | 0.000 | 54 | 5.0 | 2.2 | 1112.6 | 646.5 |
| WEEK | 6728-26-3 | 0.348 | -0.590 | 0.000 | 54 | 5.0 | 2.2 | 8666.5 | 3359.7 |
| WEEK | TOTAL VOLATILES | 0.338 | -0.581 | 0.000 | 54 | 5.0 | 2.2 | 15814.0 | 5238.5 |
| WEEK | 5881-17-4 | 0.315 | -0.561 | 0.000 | 54 | 5.0 | 2.2 | 6.2 | 2.5 |
| WEEK | 105-54-4 | 0.294 | -0.542 | 0.000 | 54 | 5.0 | 2.2 | 42.0 | 17.1 |
| WEEK | TOTAL SUGAR | 0.287 | -0.536 | 0.000 | 54 | 5.0 | 2.2 | 4473.9 | 1037.2 |
| WEEK | 124-19-6 | 0.264 | -0.513 | 0.000 | 54 | 5.0 | 2.2 | 8.5 | 7.5 |
| WEEK | 3913-81-3 | 0.252 | -0.502 | 0.000 | 54 | 5.0 | 2.2 | 1.9 | 1.4 |
| WEEK | 96-22-0 | 0.242 | -0.492 | 0.000 | 54 | 5.0 | 2.2 | 51.2 | 18.6 |
| WEEK | 2305-05-7 | 0.240 | -0.490 | 0.000 | 54 | 5.0 | 2.2 | 6.7 | 6.7 |
| WEEK | 110-93-0 | 0.179 | -0.423 | 0.001 | 54 | 5.0 | 2.2 | 2.7 | 1.5 |
| WEEK | 616-25-1 | 0.158 | -0.398 | 0.003 | 54 | 5.0 | 2.2 | 15.9 | 7.3 |
| WEEK | 75-85-4 | 0.148 | -0.385 | 0.004 | 54 | 5.0 | 2.2 | 3.9 | 2.2 |
| WEEK | 2639-63-6 | 0.141 | -0.375 | 0.005 | 54 | 5.0 | 2.2 | 10.8 | 13.0 |
| WEEK | 116-53-0 | 0.139 | -0.372 | 0.006 | 54 | 5.0 | 2.2 | 19.7 | 16.0 |
| WEEK | 142-92-7 | 0.137 | -0.370 | 0.006 | 54 | 5.0 | 2.2 | 53.5 | 49.7 |
| WEEK | 111-71-7 | 0.134 | -0.366 | 0.006 | 54 | 5.0 | 2.2 | 3.2 | 2.3 |
| WEEK | TEXTURE LIKING | 0.132 | -0.364 | 0.007 | 54 | 5.0 | 2.2 | 23.8 | 5.7 |
| WEEK | L* int | 0.127 | -0.357 | 0.008 | 54 | 5.0 | 2.2 | 54.9 | 5.8 |
| WEEK | 60415-61-4 | 0.118 | -0.343 | 0.011 | 54 | 5.0 | 2.2 | 0.7 | 1.6 |
| WEEK | 5454-09-1 | 0.112 | -0.335 | 0.013 | 54 | 5.0 | 2.2 | 3.7 | 6.2 |
| WEEK | 66-25-1 | 0.106 | -0.325 | 0.017 | 54 | 5.0 | 2.2 | 2545.9 | 1722.0 |
| WEEK | 106-32-1 | 0.101 | 0.317 | 0.019 | 54 | 5.0 | 2.2 | 2.2 | 3.0 |
| WEEK | 7452-79-1 | 0.098 | -0.312 | 0.021 | 54 | 5.0 | 2.2 | 50.0 | 31.0 |
| WEEK | 109-21-7 | 0.097 | -0.311 | 0.022 | 54 | 5.0 | 2.2 | 72.1 | 157.2 |
| WEEK | 4077-47-8 | 0.089 | -0.298 | 0.028 | 54 | 5.0 | 2.2 | 11.7 | 8.3 |
| WEEK | 123-86-4 | 0.087 | -0.296 | 0.030 | 54 | 5.0 | 2.2 | 73.5 | 85.0 |
| WEEK | 1534-08-3 | 0.082 | -0.286 | 0.036 | 54 | 5.0 | 2.2 | 0.4 | 0.2 |
| WEEK | 638-11-9 | 0.078 | -0.280 | 0.041 | 54 | 5.0 | 2.2 | 72.7 | 67.2 |
| WEEK | 628-63-7 | 0.071 | -0.266 | 0.052 | 54 | 5.0 | 2.2 | 4.6 | 1.7 |
| WEEK | A* ext | 0.070 | -0.265 | 0.053 | 54 | 5.0 | 2.2 | 36.4 | 3.1 |
| WEEK | 40716-66-3 | 0.069 | -0.262 | 0.055 | 54 | 5.0 | 2.2 | 84.3 | 107.5 |
| WEEK | GLUCOSE | 0.064 | -0.254 | 0.064 | 54 | 5.0 | 2.2 | 1594.6 | 378.2 |
| WEEK | 110-43-0 | 0.060 | -0.244 | 0.075 | 54 | 5.0 | 2.2 | 14.8 | 19.6 |
| WEEK | 53398-83-7 | 0.050 | -0.223 | 0.105 | 54 | 5.0 | 2.2 | 5.0 | 4.6 |
| WEEK | 110-39-4 | 0.049 | -0.222 | 0.107 | 54 | 5.0 | 2.2 | 40.8 | 70.0 |
| WEEK | 109-60-4 | 0.044 | 0.210 | 0.128 | 54 | 5.0 | 2.2 | 3.8 | 2.7 |
| WEEK | 564-94-3 | 0.043 | -0.208 | 0.132 | 54 | 5.0 | 2.2 | 6.5 | 7.4 |
| WEEK | 591-78-6 | 0.043 | -0.207 | 0.134 | 54 | 5.0 | 2.2 | 10.3 | 13.9 |
| WEEK | FRUCTOSE | 0.041 | -0.203 | 0.141 | 54 | 5.0 | 2.2 | 1766.7 | 381.5 |
| WEEK | TA | 0.041 | -0.201 | 0.144 | 54 | 5.0 | 2.2 | 0.8 | 0.1 |
| WEEK | 706-14-9 | 0.040 | -0.200 | 0.146 | 54 | 5.0 | 2.2 | 44.5 | 81.1 |
| WEEK | 5989-33-3 | 0.040 | -0.199 | 0.149 | 54 | 5.0 | 2.2 | 2.8 | 2.4 |
| WEEK | 104-76-7 | 0.038 | -0.195 | 0.158 | 54 | 5.0 | 2.2 | 6.0 | 4.9 |
| WEEK | 2548-87-0 | 0.036 | -0.191 | 0.167 | 54 | 5.0 | 2.2 | 2.4 | 1.5 |
| WEEK | 15111-96-3 | 0.030 | -0.173 | 0.212 | 54 | 5.0 | 2.2 | 1.2 | 1.2 |
| WEEK | 109-19-3 | 0.029 | -0.171 | 0.217 | 54 | 5.0 | 2.2 | 2.7 | 3.8 |
| WEEK | pH | 0.029 | -0.169 | 0.221 | 54 | 5.0 | 2.2 | 3.7 | 0.2 |
| WEEK | 108-10-1 | 0.028 | 0.167 | 0.228 | 54 | 5.0 | 2.2 | 1.5 | 2.6 |
| WEEK | 10522-34-6 | 0.025 | -0.159 | 0.252 | 54 | 5.0 | 2.2 | 1.1 | 1.1 |
| WEEK | L* ext | 0.025 | -0.158 | 0.255 | 54 | 5.0 | 2.2 | 33.6 | 2.6 |
| WEEK | A* int | 0.024 | 0.156 | 0.261 | 54 | 5.0 | 2.2 | 28.8 | 7.6 |
| WEEK | 134-20-3 | 0.024 | 0.156 | 0.261 | 54 | 5.0 | 2.2 | 0.1 | 0.7 |
| WEEK | 123-66-0 | 0.021 | 0.146 | 0.292 | 54 | 5.0 | 2.2 | 108.2 | 128.0 |
| WEEK | 623-42-7 | 0.019 | -0.139 | 0.316 | 54 | 5.0 | 2.2 | 2780.2 | 1376.8 |
| WEEK | FORCE | 0.019 | -0.139 | 0.316 | 54 | 5.0 | 2.2 | 0.6 | 0.2 |
| WEEK | 4887-30-3 | 0.019 | -0.138 | 0.319 | 54 | 5.0 | 2.2 | 16.9 | 32.5 |
| WEEK | 123-92-2 | 0.018 | 0.132 | 0.340 | 54 | 5.0 | 2.2 | 23.0 | 21.5 |
| WEEK | 96-04-8 | 0.016 | -0.127 | 0.359 | 54 | 5.0 | 2.2 | 3.1 | 8.0 |
| WEEK | 821-55-6 | 0.015 | -0.123 | 0.374 | 54 | 5.0 | 2.2 | 3.5 | 8.2 |
| WEEK | 539-82-2 | 0.012 | -0.111 | 0.425 | 54 | 5.0 | 2.2 | 3.3 | 3.9 |
| WEEK | 540-18-1 | 0.012 | -0.108 | 0.435 | 54 | 5.0 | 2.2 | 3.5 | 3.2 |
| WEEK | 128-37-0 | 0.010 | 0.099 | 0.475 | 54 | 5.0 | 2.2 | 4.1 | 3.7 |
| WEEK | 55514-48-2 | 0.009 | 0.097 | 0.485 | 54 | 5.0 | 2.2 | 0.5 | 0.5 |
| WEEK | 124-13-0 | 0.009 | -0.096 | 0.491 | 54 | 5.0 | 2.2 | 5.9 | 3.0 |
| WEEK | 112-14-1 | 0.009 | -0.095 | 0.495 | 54 | 5.0 | 2.2 | 18.3 | 24.0 |
| WEEK | CITRIC ACID | 0.009 | -0.094 | 0.499 | 54 | 5.0 | 2.2 | 741.0 | 147.2 |
| WEEK | SOURNESS INTENSITY | 0.009 | 0.093 | 0.505 | 54 | 5.0 | 2.2 | 18.1 | 3.1 |
| WEEK | 623-43-8 | 0.008 | 0.090 | 0.518 | 54 | 5.0 | 2.2 | 3.4 | 3.4 |
| WEEK | 105-66-8 | 0.008 | -0.088 | 0.528 | 54 | 5.0 | 2.2 | 5.0 | 3.7 |
| WEEK | B* int | 0.007 | 0.084 | 0.544 | 54 | 5.0 | 2.2 | 25.8 | 4.5 |
| WEEK | 110-62-3 | 0.005 | -0.074 | 0.597 | 54 | 5.0 | 2.2 | 7.9 | 8.9 |
| WEEK | 1576-95-0 | 0.005 | -0.072 | 0.604 | 54 | 5.0 | 2.2 | 2.1 | 2.0 |
| WEEK | B* ext | 0.005 | -0.070 | 0.613 | 54 | 5.0 | 2.2 | 19.0 | 3.3 |
| WEEK | 2311-46-8 | 0.004 | -0.066 | 0.637 | 54 | 5.0 | 2.2 | 3.9 | 4.4 |
| WEEK | MALIC ACID | 0.004 | -0.065 | 0.641 | 54 | 5.0 | 2.2 | 212.4 | 51.6 |
| WEEK | 29811-50-5 | 0.004 | -0.064 | 0.643 | 54 | 5.0 | 2.2 | 3.2 | 5.3 |
| WEEK | 103-09-3 | 0.003 | 0.057 | 0.680 | 54 | 5.0 | 2.2 | 3.0 | 1.1 |
| WEEK | 140-11-4 | 0.003 | -0.054 | 0.700 | 54 | 5.0 | 2.2 | 11.1 | 8.6 |
| WEEK | 2432-51-1 | 0.002 | 0.048 | 0.729 | 54 | 5.0 | 2.2 | 4.4 | 5.8 |
| WEEK | 556-24-1 | 0.002 | -0.044 | 0.752 | 54 | 5.0 | 2.2 | 46.6 | 57.0 |
| WEEK | 78-70-6 | 0.001 | -0.038 | 0.787 | 54 | 5.0 | 2.2 | 128.8 | 113.0 |
| WEEK | 589-38-8 | 0.001 | 0.036 | 0.798 | 54 | 5.0 | 2.2 | 1.9 | 1.2 |
| WEEK | 2497-18-9 | 0.001 | 0.033 | 0.815 | 54 | 5.0 | 2.2 | 24.9 | 21.5 |
| WEEK | 1191-16-8 | 0.001 | 0.029 | 0.837 | 54 | 5.0 | 2.2 | 5.5 | 7.0 |
| WEEK | 928-95-0 | 0.001 | 0.028 | 0.838 | 54 | 5.0 | 2.2 | 66.8 | 61.7 |
| WEEK | 106-70-7 | 0.001 | -0.027 | 0.845 | 54 | 5.0 | 2.2 | 252.7 | 164.0 |
| WEEK | 71-41-0 | 0.001 | -0.026 | 0.854 | 54 | 5.0 | 2.2 | 1.0 | 1.3 |
| WEEK | 20664-46-4 | 0.000 | -0.020 | 0.887 | 54 | 5.0 | 2.2 | 20.6 | 19.0 |
| WEEK | 7786-58-5 | 0.000 | -0.019 | 0.890 | 54 | 5.0 | 2.2 | 12.2 | 28.4 |
| WEEK | 29674-47-3 | 0.000 | -0.019 | 0.892 | 54 | 5.0 | 2.2 | 5.0 | 5.0 |
| WEEK | 110-38-3 | 0.000 | -0.019 | 0.893 | 54 | 5.0 | 2.2 | 2.0 | 2.6 |
| WEEK | 105-37-3 | 0.000 | 0.017 | 0.903 | 54 | 5.0 | 2.2 | 10.1 | 14.1 |
| WEEK | 624-41-9 | 0.000 | -0.010 | 0.945 | 54 | 5.0 | 2.2 | 18.9 | 18.1 |
| WEEK | 624-24-8 | 0.000 | -0.007 | 0.960 | 54 | 5.0 | 2.2 | 5.6 | 3.7 |
| WEEK | 111-27-3 | 0.000 | 0.004 | 0.976 | 54 | 5.0 | 2.2 | 45.5 | 94.6 |

Regression of harvest week during season (X) on panel responses and metabolite concentration (Y). Coefficient of determination (R^2^), correlation coefficient, p-value, sample size (n), mean and standard deviation of X and Y derived from bivariate fit in JMP 8.
