# Supplementary material for: Strawberry Flavor: Diverse Chemical Compositions, a Seasonal Influence, and Effects on Sensory Perception
Source: PLoS One. 2014 Feb 11;9(2):e88446. doi: 10.1371/journal.pone.0088446 (PMC3921181; doi:10.1371/journal.pone.0088446)
Supplement: Table S4 — Fruit quality bivariate fit. Regression of chemical and physical measures of fruit (X) to panel responses (Y). Coefficient of determination (R2), correlation coefficient, p-value, sample size (n), mean and standard deviation of X and Y derived from bivariate fit in JMP 8. (DOCX) [file pone.0088446.s007.docx]

**Table S4. Fruit quality bivariate fit.**

| **X** | **Y** | **R^2^** | **CORR COEFF** | **p-VALUE** | **n** | **MEAN X** | **STD DEV X** | **MEAN Y** | **STD DEV Y** |
| --- | --- | --- | --- | --- | --- | --- | --- | --- | --- |
| TOTAL SUGAR | OVERALL LIKING | 0.489 | 0.699 | 0.000 | 54 | 4473.9 | 1037.2 | 23.8 | 5.7 |
| SSC | OVERALL LIKING | 0.457 | 0.676 | 0.000 | 54 | 7.4 | 1.4 | 23.8 | 5.7 |
| SUCROSE | OVERALL LIKING | 0.442 | 0.665 | 0.000 | 54 | 1112.6 | 646.5 | 23.8 | 5.7 |
| 1629-58-9 | OVERALL LIKING | 0.437 | 0.661 | 0.000 | 54 | 117.9 | 65.9 | 23.8 | 5.7 |
| 1576-87-0 | OVERALL LIKING | 0.371 | 0.609 | 0.000 | 54 | 37.5 | 20.1 | 23.8 | 5.7 |
| 2305-05-7 | OVERALL LIKING | 0.310 | 0.557 | 0.000 | 54 | 6.7 | 6.7 | 23.8 | 5.7 |
| 1576-86-9 | OVERALL LIKING | 0.301 | 0.549 | 0.000 | 54 | 37.8 | 21.7 | 23.8 | 5.7 |
| 111-71-7 | OVERALL LIKING | 0.288 | 0.537 | 0.000 | 54 | 3.2 | 2.3 | 23.8 | 5.7 |
| 540-18-1 | OVERALL LIKING | 0.244 | 0.494 | 0.000 | 54 | 3.5 | 3.2 | 23.8 | 5.7 |
| 3913-81-3 | OVERALL LIKING | 0.241 | 0.491 | 0.000 | 54 | 1.9 | 1.4 | 23.8 | 5.7 |
| 110-93-0 | OVERALL LIKING | 0.228 | 0.477 | 0.000 | 54 | 2.7 | 1.5 | 23.8 | 5.7 |
| 2639-63-6 | OVERALL LIKING | 0.200 | 0.447 | 0.001 | 54 | 10.8 | 13.0 | 23.8 | 5.7 |
| 124-19-6 | OVERALL LIKING | 0.196 | 0.443 | 0.001 | 54 | 8.5 | 7.5 | 23.8 | 5.7 |
| 2548-87-0 | OVERALL LIKING | 0.191 | 0.437 | 0.001 | 54 | 2.4 | 1.5 | 23.8 | 5.7 |
| 4077-47-8 | OVERALL LIKING | 0.189 | 0.434 | 0.001 | 54 | 11.7 | 8.3 | 23.8 | 5.7 |
| 104-76-7 | OVERALL LIKING | 0.187 | 0.432 | 0.001 | 54 | 6.0 | 4.9 | 23.8 | 5.7 |
| TOTAL VOLATILES | OVERALL LIKING | 0.179 | 0.424 | 0.001 | 54 | 15814.0 | 5238.5 | 23.8 | 5.7 |
| 638-11-9 | OVERALL LIKING | 0.179 | 0.423 | 0.001 | 54 | 72.7 | 67.2 | 23.8 | 5.7 |
| 60415-61-4 | OVERALL LIKING | 0.177 | 0.421 | 0.002 | 54 | 0.7 | 1.6 | 23.8 | 5.7 |
| GLUCOSE | OVERALL LIKING | 0.175 | 0.419 | 0.002 | 54 | 1594.6 | 378.2 | 23.8 | 5.7 |
| 5989-33-3 | OVERALL LIKING | 0.168 | 0.410 | 0.002 | 54 | 2.8 | 2.4 | 23.8 | 5.7 |
| 40716-66-3 | OVERALL LIKING | 0.164 | 0.405 | 0.002 | 54 | 84.3 | 107.5 | 23.8 | 5.7 |
| 5881-17-4 | OVERALL LIKING | 0.157 | 0.397 | 0.003 | 54 | 6.2 | 2.5 | 23.8 | 5.7 |
| 109-19-3 | OVERALL LIKING | 0.154 | 0.392 | 0.003 | 54 | 2.7 | 3.8 | 23.8 | 5.7 |
| L* int | OVERALL LIKING | 0.147 | 0.384 | 0.004 | 54 | 54.9 | 5.8 | 23.8 | 5.7 |
| 109-21-7 | OVERALL LIKING | 0.143 | 0.378 | 0.005 | 54 | 72.1 | 157.2 | 23.8 | 5.7 |
| 142-92-7 | OVERALL LIKING | 0.138 | 0.371 | 0.006 | 54 | 53.5 | 49.7 | 23.8 | 5.7 |
| 110-43-0 | OVERALL LIKING | 0.137 | 0.370 | 0.006 | 54 | 14.8 | 19.6 | 23.8 | 5.7 |
| FRUCTOSE | OVERALL LIKING | 0.129 | 0.359 | 0.008 | 54 | 1766.7 | 381.5 | 23.8 | 5.7 |
| 5454-09-1 | OVERALL LIKING | 0.125 | 0.353 | 0.009 | 54 | 3.7 | 6.2 | 23.8 | 5.7 |
| 706-14-9 | OVERALL LIKING | 0.122 | 0.349 | 0.010 | 54 | 44.5 | 81.1 | 23.8 | 5.7 |
| 591-78-6 | OVERALL LIKING | 0.118 | 0.343 | 0.011 | 54 | 10.3 | 13.9 | 23.8 | 5.7 |
| 123-86-4 | OVERALL LIKING | 0.108 | 0.329 | 0.015 | 54 | 73.5 | 85.0 | 23.8 | 5.7 |
| 6728-26-3 | OVERALL LIKING | 0.108 | 0.329 | 0.015 | 54 | 8666.5 | 3359.7 | 23.8 | 5.7 |
| 53398-83-7 | OVERALL LIKING | 0.106 | 0.325 | 0.016 | 54 | 5.0 | 4.6 | 23.8 | 5.7 |
| A* int | OVERALL LIKING | 0.105 | -0.324 | 0.017 | 54 | 28.8 | 7.6 | 23.8 | 5.7 |
| TA | OVERALL LIKING | 0.099 | 0.314 | 0.021 | 54 | 0.8 | 0.1 | 23.8 | 5.7 |
| 110-38-3 | OVERALL LIKING | 0.094 | 0.307 | 0.024 | 54 | 2.0 | 2.6 | 23.8 | 5.7 |
| 105-66-8 | OVERALL LIKING | 0.094 | 0.306 | 0.024 | 54 | 5.0 | 3.7 | 23.8 | 5.7 |
| 105-54-4 | OVERALL LIKING | 0.088 | 0.297 | 0.029 | 54 | 42.0 | 17.1 | 23.8 | 5.7 |
| 616-25-1 | OVERALL LIKING | 0.082 | 0.286 | 0.036 | 54 | 15.9 | 7.3 | 23.8 | 5.7 |
| 96-22-0 | OVERALL LIKING | 0.079 | 0.280 | 0.040 | 54 | 51.2 | 18.6 | 23.8 | 5.7 |
| 134-20-3 | OVERALL LIKING | 0.076 | -0.276 | 0.043 | 54 | 0.1 | 0.7 | 23.8 | 5.7 |
| 10522-34-6 | OVERALL LIKING | 0.076 | 0.276 | 0.044 | 54 | 1.1 | 1.1 | 23.8 | 5.7 |
| 623-42-7 | OVERALL LIKING | 0.075 | 0.275 | 0.045 | 54 | 2780.2 | 1376.8 | 23.8 | 5.7 |
| 1191-16-8 | OVERALL LIKING | 0.072 | -0.268 | 0.050 | 54 | 5.5 | 7.0 | 23.8 | 5.7 |
| 110-39-4 | OVERALL LIKING | 0.067 | 0.258 | 0.059 | 54 | 40.8 | 70.0 | 23.8 | 5.7 |
| 55514-48-2 | OVERALL LIKING | 0.065 | -0.254 | 0.064 | 54 | 0.5 | 0.5 | 23.8 | 5.7 |
| 2311-46-8 | OVERALL LIKING | 0.061 | 0.247 | 0.072 | 54 | 3.9 | 4.4 | 23.8 | 5.7 |
| B* int | OVERALL LIKING | 0.059 | -0.242 | 0.078 | 54 | 25.8 | 4.5 | 23.8 | 5.7 |
| 116-53-0 | OVERALL LIKING | 0.058 | 0.240 | 0.080 | 54 | 19.7 | 16.0 | 23.8 | 5.7 |
| CITRIC ACID | OVERALL LIKING | 0.056 | 0.237 | 0.084 | 54 | 741.0 | 147.2 | 23.8 | 5.7 |
| pH | OVERALL LIKING | 0.053 | 0.231 | 0.094 | 54 | 3.7 | 0.2 | 23.8 | 5.7 |
| 29674-47-3 | OVERALL LIKING | 0.051 | 0.226 | 0.100 | 54 | 5.0 | 5.0 | 23.8 | 5.7 |
| 4887-30-3 | OVERALL LIKING | 0.051 | 0.226 | 0.101 | 54 | 16.9 | 32.5 | 23.8 | 5.7 |
| 564-94-3 | OVERALL LIKING | 0.047 | 0.218 | 0.114 | 54 | 6.5 | 7.4 | 23.8 | 5.7 |
| 623-43-8 | OVERALL LIKING | 0.041 | -0.203 | 0.142 | 54 | 3.4 | 3.4 | 23.8 | 5.7 |
| 539-82-2 | OVERALL LIKING | 0.039 | 0.196 | 0.155 | 54 | 3.3 | 3.9 | 23.8 | 5.7 |
| 66-25-1 | OVERALL LIKING | 0.035 | 0.187 | 0.175 | 54 | 2545.9 | 1722.0 | 23.8 | 5.7 |
| 628-63-7 | OVERALL LIKING | 0.035 | 0.187 | 0.177 | 54 | 4.6 | 1.7 | 23.8 | 5.7 |
| FORCE | OVERALL LIKING | 0.034 | 0.185 | 0.181 | 54 | 0.6 | 0.2 | 23.8 | 5.7 |
| 7786-58-5 | OVERALL LIKING | 0.033 | 0.182 | 0.188 | 54 | 12.2 | 28.4 | 23.8 | 5.7 |
| A* ext | OVERALL LIKING | 0.033 | 0.182 | 0.189 | 54 | 36.4 | 3.1 | 23.8 | 5.7 |
| 71-41-0 | OVERALL LIKING | 0.029 | 0.172 | 0.215 | 54 | 1.0 | 1.3 | 23.8 | 5.7 |
| 624-41-9 | OVERALL LIKING | 0.028 | -0.168 | 0.225 | 54 | 18.9 | 18.1 | 23.8 | 5.7 |
| 109-60-4 | OVERALL LIKING | 0.027 | -0.166 | 0.231 | 54 | 3.8 | 2.7 | 23.8 | 5.7 |
| MALIC ACID | OVERALL LIKING | 0.027 | 0.165 | 0.234 | 54 | 212.4 | 51.6 | 23.8 | 5.7 |
| 111-27-3 | OVERALL LIKING | 0.027 | -0.164 | 0.236 | 54 | 45.5 | 94.6 | 23.8 | 5.7 |
| 124-13-0 | OVERALL LIKING | 0.024 | 0.154 | 0.267 | 54 | 5.9 | 3.0 | 23.8 | 5.7 |
| 78-70-6 | OVERALL LIKING | 0.023 | 0.153 | 0.270 | 54 | 128.8 | 113.0 | 23.8 | 5.7 |
| 821-55-6 | OVERALL LIKING | 0.022 | 0.148 | 0.286 | 54 | 3.5 | 8.2 | 23.8 | 5.7 |
| 624-24-8 | OVERALL LIKING | 0.022 | 0.147 | 0.289 | 54 | 5.6 | 3.7 | 23.8 | 5.7 |
| 7452-79-1 | OVERALL LIKING | 0.022 | 0.147 | 0.289 | 54 | 50.0 | 31.0 | 23.8 | 5.7 |
| 106-70-7 | OVERALL LIKING | 0.021 | 0.145 | 0.296 | 54 | 252.7 | 164.0 | 23.8 | 5.7 |
| 112-14-1 | OVERALL LIKING | 0.021 | 0.144 | 0.298 | 54 | 18.3 | 24.0 | 23.8 | 5.7 |
| 1534-08-3 | OVERALL LIKING | 0.018 | 0.135 | 0.332 | 54 | 0.4 | 0.2 | 23.8 | 5.7 |
| 20664-46-4 | OVERALL LIKING | 0.018 | 0.133 | 0.339 | 54 | 20.6 | 19.0 | 23.8 | 5.7 |
| 589-38-8 | OVERALL LIKING | 0.018 | 0.133 | 0.340 | 54 | 1.9 | 1.2 | 23.8 | 5.7 |
| 123-66-0 | OVERALL LIKING | 0.017 | 0.132 | 0.341 | 54 | 108.2 | 128.0 | 23.8 | 5.7 |
| 2432-51-1 | OVERALL LIKING | 0.016 | 0.127 | 0.360 | 54 | 4.4 | 5.8 | 23.8 | 5.7 |
| 75-85-4 | OVERALL LIKING | 0.014 | 0.119 | 0.390 | 54 | 3.9 | 2.2 | 23.8 | 5.7 |
| 928-95-0 | OVERALL LIKING | 0.012 | -0.111 | 0.422 | 54 | 66.8 | 61.7 | 23.8 | 5.7 |
| 140-11-4 | OVERALL LIKING | 0.012 | -0.111 | 0.423 | 54 | 11.1 | 8.6 | 23.8 | 5.7 |
| 103-09-3 | OVERALL LIKING | 0.011 | -0.106 | 0.446 | 54 | 3.0 | 1.1 | 23.8 | 5.7 |
| 123-92-2 | OVERALL LIKING | 0.010 | -0.099 | 0.475 | 54 | 23.0 | 21.5 | 23.8 | 5.7 |
| B* ext | OVERALL LIKING | 0.009 | -0.095 | 0.492 | 54 | 19.0 | 3.3 | 23.8 | 5.7 |
| 128-37-0 | OVERALL LIKING | 0.007 | 0.086 | 0.536 | 54 | 4.1 | 3.7 | 23.8 | 5.7 |
| 2497-18-9 | OVERALL LIKING | 0.006 | -0.075 | 0.590 | 54 | 24.9 | 21.5 | 23.8 | 5.7 |
| 556-24-1 | OVERALL LIKING | 0.004 | 0.065 | 0.638 | 54 | 46.6 | 57.0 | 23.8 | 5.7 |
| 29811-50-5 | OVERALL LIKING | 0.004 | -0.062 | 0.658 | 54 | 3.2 | 5.3 | 23.8 | 5.7 |
| 110-62-3 | OVERALL LIKING | 0.003 | 0.057 | 0.680 | 54 | 7.9 | 8.9 | 23.8 | 5.7 |
| 96-04-8 | OVERALL LIKING | 0.003 | -0.052 | 0.711 | 54 | 3.1 | 8.0 | 23.8 | 5.7 |
| 1576-95-0 | OVERALL LIKING | 0.002 | 0.040 | 0.773 | 54 | 2.1 | 2.0 | 23.8 | 5.7 |
| 106-32-1 | OVERALL LIKING | 0.001 | -0.032 | 0.817 | 54 | 2.2 | 3.0 | 23.8 | 5.7 |
| 108-10-1 | OVERALL LIKING | 0.001 | -0.023 | 0.870 | 54 | 1.5 | 2.6 | 23.8 | 5.7 |
| 15111-96-3 | OVERALL LIKING | 0.000 | 0.018 | 0.897 | 54 | 1.2 | 1.2 | 23.8 | 5.7 |
| 105-37-3 | OVERALL LIKING | 0.000 | -0.009 | 0.946 | 54 | 10.1 | 14.1 | 23.8 | 5.7 |
| L* ext | OVERALL LIKING | 0.000 | 0.004 | 0.976 | 54 | 33.6 | 2.6 | 23.8 | 5.7 |
| FORCE | TEXTURE LIKING | 0.358 | 0.598 | 0.000 | 54 | 0.6 | 0.2 | 23.8 | 5.7 |
| MALIC ACID | TEXTURE LIKING | 0.282 | 0.531 | 0.000 | 54 | 212.4 | 51.6 | 23.8 | 5.7 |
| 134-20-3 | TEXTURE LIKING | 0.277 | -0.526 | 0.000 | 54 | 0.1 | 0.7 | 23.8 | 5.7 |
| 104-76-7 | TEXTURE LIKING | 0.231 | 0.480 | 0.000 | 54 | 6.0 | 4.9 | 23.8 | 5.7 |
| 623-43-8 | TEXTURE LIKING | 0.179 | -0.423 | 0.001 | 54 | 3.4 | 3.4 | 23.8 | 5.7 |
| 1629-58-9 | TEXTURE LIKING | 0.151 | 0.388 | 0.004 | 54 | 117.9 | 65.9 | 23.8 | 5.7 |
| 103-09-3 | TEXTURE LIKING | 0.145 | -0.381 | 0.004 | 54 | 3.0 | 1.1 | 23.8 | 5.7 |
| 1576-87-0 | TEXTURE LIKING | 0.145 | 0.381 | 0.005 | 54 | 37.5 | 20.1 | 23.8 | 5.7 |
| 638-11-9 | TEXTURE LIKING | 0.141 | 0.375 | 0.005 | 54 | 72.7 | 67.2 | 23.8 | 5.7 |
| 40716-66-3 | TEXTURE LIKING | 0.132 | 0.364 | 0.007 | 54 | 84.3 | 107.5 | 23.8 | 5.7 |
| 2548-87-0 | TEXTURE LIKING | 0.129 | 0.360 | 0.008 | 54 | 2.4 | 1.5 | 23.8 | 5.7 |
| 111-71-7 | TEXTURE LIKING | 0.122 | 0.349 | 0.010 | 54 | 3.2 | 2.3 | 23.8 | 5.7 |
| 110-38-3 | TEXTURE LIKING | 0.117 | 0.342 | 0.011 | 54 | 2.0 | 2.6 | 23.8 | 5.7 |
| 2305-05-7 | TEXTURE LIKING | 0.116 | 0.341 | 0.012 | 54 | 6.7 | 6.7 | 23.8 | 5.7 |
| 60415-61-4 | TEXTURE LIKING | 0.104 | 0.322 | 0.018 | 54 | 0.7 | 1.6 | 23.8 | 5.7 |
| SUCROSE | TEXTURE LIKING | 0.102 | 0.320 | 0.018 | 54 | 1112.6 | 646.5 | 23.8 | 5.7 |
| 556-24-1 | TEXTURE LIKING | 0.097 | -0.312 | 0.022 | 54 | 46.6 | 57.0 | 23.8 | 5.7 |
| 123-92-2 | TEXTURE LIKING | 0.090 | -0.300 | 0.027 | 54 | 23.0 | 21.5 | 23.8 | 5.7 |
| 5454-09-1 | TEXTURE LIKING | 0.080 | 0.282 | 0.039 | 54 | 3.7 | 6.2 | 23.8 | 5.7 |
| 5989-33-3 | TEXTURE LIKING | 0.078 | 0.280 | 0.040 | 54 | 2.8 | 2.4 | 23.8 | 5.7 |
| 110-62-3 | TEXTURE LIKING | 0.076 | 0.276 | 0.043 | 54 | 7.9 | 8.9 | 23.8 | 5.7 |
| 55514-48-2 | TEXTURE LIKING | 0.076 | -0.275 | 0.044 | 54 | 0.5 | 0.5 | 23.8 | 5.7 |
| 109-21-7 | TEXTURE LIKING | 0.067 | 0.260 | 0.058 | 54 | 72.1 | 157.2 | 23.8 | 5.7 |
| TOTAL SUGAR | TEXTURE LIKING | 0.067 | 0.260 | 0.058 | 54 | 4473.9 | 1037.2 | 23.8 | 5.7 |
| 124-19-6 | TEXTURE LIKING | 0.065 | 0.256 | 0.062 | 54 | 8.5 | 7.5 | 23.8 | 5.7 |
| 110-93-0 | TEXTURE LIKING | 0.065 | 0.256 | 0.062 | 54 | 2.7 | 1.5 | 23.8 | 5.7 |
| 540-18-1 | TEXTURE LIKING | 0.063 | 0.251 | 0.068 | 54 | 3.5 | 3.2 | 23.8 | 5.7 |
| 105-66-8 | TEXTURE LIKING | 0.062 | 0.250 | 0.068 | 54 | 5.0 | 3.7 | 23.8 | 5.7 |
| 110-43-0 | TEXTURE LIKING | 0.062 | 0.249 | 0.070 | 54 | 14.8 | 19.6 | 23.8 | 5.7 |
| 109-60-4 | TEXTURE LIKING | 0.059 | -0.244 | 0.076 | 54 | 3.8 | 2.7 | 23.8 | 5.7 |
| 1576-86-9 | TEXTURE LIKING | 0.058 | 0.241 | 0.079 | 54 | 37.8 | 21.7 | 23.8 | 5.7 |
| 4077-47-8 | TEXTURE LIKING | 0.058 | 0.241 | 0.080 | 54 | 11.7 | 8.3 | 23.8 | 5.7 |
| pH | TEXTURE LIKING | 0.056 | 0.237 | 0.084 | 54 | 3.7 | 0.2 | 23.8 | 5.7 |
| SSC | TEXTURE LIKING | 0.055 | 0.234 | 0.089 | 54 | 7.4 | 1.4 | 23.8 | 5.7 |
| 10522-34-6 | TEXTURE LIKING | 0.048 | 0.218 | 0.113 | 54 | 1.1 | 1.1 | 23.8 | 5.7 |
| 109-19-3 | TEXTURE LIKING | 0.045 | 0.213 | 0.123 | 54 | 2.7 | 3.8 | 23.8 | 5.7 |
| 706-14-9 | TEXTURE LIKING | 0.044 | 0.210 | 0.128 | 54 | 44.5 | 81.1 | 23.8 | 5.7 |
| 2639-63-6 | TEXTURE LIKING | 0.040 | 0.200 | 0.147 | 54 | 10.8 | 13.0 | 23.8 | 5.7 |
| 111-27-3 | TEXTURE LIKING | 0.039 | -0.199 | 0.150 | 54 | 45.5 | 94.6 | 23.8 | 5.7 |
| 2432-51-1 | TEXTURE LIKING | 0.035 | 0.187 | 0.176 | 54 | 4.4 | 5.8 | 23.8 | 5.7 |
| 564-94-3 | TEXTURE LIKING | 0.035 | 0.186 | 0.177 | 54 | 6.5 | 7.4 | 23.8 | 5.7 |
| 110-39-4 | TEXTURE LIKING | 0.034 | 0.184 | 0.183 | 54 | 40.8 | 70.0 | 23.8 | 5.7 |
| 3913-81-3 | TEXTURE LIKING | 0.034 | 0.184 | 0.183 | 54 | 1.9 | 1.4 | 23.8 | 5.7 |
| 539-82-2 | TEXTURE LIKING | 0.030 | 0.173 | 0.212 | 54 | 3.3 | 3.9 | 23.8 | 5.7 |
| 624-24-8 | TEXTURE LIKING | 0.028 | 0.168 | 0.226 | 54 | 5.6 | 3.7 | 23.8 | 5.7 |
| 116-53-0 | TEXTURE LIKING | 0.026 | 0.163 | 0.240 | 54 | 19.7 | 16.0 | 23.8 | 5.7 |
| TA | TEXTURE LIKING | 0.026 | 0.161 | 0.244 | 54 | 0.8 | 0.1 | 23.8 | 5.7 |
| 123-86-4 | TEXTURE LIKING | 0.026 | 0.160 | 0.247 | 54 | 73.5 | 85.0 | 23.8 | 5.7 |
| TOTAL VOLATILES | TEXTURE LIKING | 0.025 | 0.158 | 0.254 | 54 | 15814.0 | 5238.5 | 23.8 | 5.7 |
| 5881-17-4 | TEXTURE LIKING | 0.025 | 0.157 | 0.256 | 54 | 6.2 | 2.5 | 23.8 | 5.7 |
| 66-25-1 | TEXTURE LIKING | 0.023 | 0.153 | 0.269 | 54 | 2545.9 | 1722.0 | 23.8 | 5.7 |
| 78-70-6 | TEXTURE LIKING | 0.023 | 0.152 | 0.272 | 54 | 128.8 | 113.0 | 23.8 | 5.7 |
| 124-13-0 | TEXTURE LIKING | 0.023 | -0.152 | 0.273 | 54 | 5.9 | 3.0 | 23.8 | 5.7 |
| 142-92-7 | TEXTURE LIKING | 0.023 | 0.151 | 0.274 | 54 | 53.5 | 49.7 | 23.8 | 5.7 |
| 2311-46-8 | TEXTURE LIKING | 0.023 | 0.151 | 0.276 | 54 | 3.9 | 4.4 | 23.8 | 5.7 |
| 928-95-0 | TEXTURE LIKING | 0.020 | -0.143 | 0.302 | 54 | 66.8 | 61.7 | 23.8 | 5.7 |
| 96-04-8 | TEXTURE LIKING | 0.019 | -0.138 | 0.319 | 54 | 3.1 | 8.0 | 23.8 | 5.7 |
| 20664-46-4 | TEXTURE LIKING | 0.019 | -0.137 | 0.324 | 54 | 20.6 | 19.0 | 23.8 | 5.7 |
| 821-55-6 | TEXTURE LIKING | 0.018 | 0.135 | 0.329 | 54 | 3.5 | 8.2 | 23.8 | 5.7 |
| 53398-83-7 | TEXTURE LIKING | 0.018 | 0.133 | 0.338 | 54 | 5.0 | 4.6 | 23.8 | 5.7 |
| 29811-50-5 | TEXTURE LIKING | 0.017 | -0.131 | 0.346 | 54 | 3.2 | 5.3 | 23.8 | 5.7 |
| 128-37-0 | TEXTURE LIKING | 0.017 | 0.129 | 0.352 | 54 | 4.1 | 3.7 | 23.8 | 5.7 |
| 140-11-4 | TEXTURE LIKING | 0.016 | -0.127 | 0.359 | 54 | 11.1 | 8.6 | 23.8 | 5.7 |
| 4887-30-3 | TEXTURE LIKING | 0.016 | 0.126 | 0.363 | 54 | 16.9 | 32.5 | 23.8 | 5.7 |
| 75-85-4 | TEXTURE LIKING | 0.015 | 0.124 | 0.371 | 54 | 3.9 | 2.2 | 23.8 | 5.7 |
| L* ext | TEXTURE LIKING | 0.015 | -0.123 | 0.377 | 54 | 33.6 | 2.6 | 23.8 | 5.7 |
| 1191-16-8 | TEXTURE LIKING | 0.015 | -0.121 | 0.385 | 54 | 5.5 | 7.0 | 23.8 | 5.7 |
| GLUCOSE | TEXTURE LIKING | 0.013 | 0.115 | 0.408 | 54 | 1594.6 | 378.2 | 23.8 | 5.7 |
| B* ext | TEXTURE LIKING | 0.013 | -0.113 | 0.417 | 54 | 19.0 | 3.3 | 23.8 | 5.7 |
| 624-41-9 | TEXTURE LIKING | 0.012 | -0.111 | 0.424 | 54 | 18.9 | 18.1 | 23.8 | 5.7 |
| 6728-26-3 | TEXTURE LIKING | 0.012 | 0.108 | 0.435 | 54 | 8666.5 | 3359.7 | 23.8 | 5.7 |
| A* ext | TEXTURE LIKING | 0.010 | 0.099 | 0.475 | 54 | 36.4 | 3.1 | 23.8 | 5.7 |
| 2497-18-9 | TEXTURE LIKING | 0.008 | -0.092 | 0.508 | 54 | 24.9 | 21.5 | 23.8 | 5.7 |
| 71-41-0 | TEXTURE LIKING | 0.008 | 0.087 | 0.530 | 54 | 1.0 | 1.3 | 23.8 | 5.7 |
| 589-38-8 | TEXTURE LIKING | 0.007 | 0.087 | 0.534 | 54 | 1.9 | 1.2 | 23.8 | 5.7 |
| 106-32-1 | TEXTURE LIKING | 0.007 | -0.085 | 0.542 | 54 | 2.2 | 3.0 | 23.8 | 5.7 |
| 616-25-1 | TEXTURE LIKING | 0.007 | 0.082 | 0.555 | 54 | 15.9 | 7.3 | 23.8 | 5.7 |
| 106-70-7 | TEXTURE LIKING | 0.006 | -0.076 | 0.587 | 54 | 252.7 | 164.0 | 23.8 | 5.7 |
| B* int | TEXTURE LIKING | 0.006 | -0.075 | 0.588 | 54 | 25.8 | 4.5 | 23.8 | 5.7 |
| 7452-79-1 | TEXTURE LIKING | 0.005 | 0.072 | 0.607 | 54 | 50.0 | 31.0 | 23.8 | 5.7 |
| 105-54-4 | TEXTURE LIKING | 0.005 | 0.068 | 0.626 | 54 | 42.0 | 17.1 | 23.8 | 5.7 |
| A* int | TEXTURE LIKING | 0.004 | -0.060 | 0.666 | 54 | 28.8 | 7.6 | 23.8 | 5.7 |
| 628-63-7 | TEXTURE LIKING | 0.003 | -0.055 | 0.693 | 54 | 4.6 | 1.7 | 23.8 | 5.7 |
| 591-78-6 | TEXTURE LIKING | 0.003 | 0.054 | 0.700 | 54 | 10.3 | 13.9 | 23.8 | 5.7 |
| 7786-58-5 | TEXTURE LIKING | 0.003 | -0.052 | 0.707 | 54 | 12.2 | 28.4 | 23.8 | 5.7 |
| FRUCTOSE | TEXTURE LIKING | 0.002 | 0.050 | 0.720 | 54 | 1766.7 | 381.5 | 23.8 | 5.7 |
| 1576-95-0 | TEXTURE LIKING | 0.002 | 0.047 | 0.736 | 54 | 2.1 | 2.0 | 23.8 | 5.7 |
| CITRIC ACID | TEXTURE LIKING | 0.002 | -0.047 | 0.736 | 54 | 741.0 | 147.2 | 23.8 | 5.7 |
| 112-14-1 | TEXTURE LIKING | 0.001 | -0.028 | 0.843 | 54 | 18.3 | 24.0 | 23.8 | 5.7 |
| 105-37-3 | TEXTURE LIKING | 0.001 | -0.026 | 0.851 | 54 | 10.1 | 14.1 | 23.8 | 5.7 |
| 29674-47-3 | TEXTURE LIKING | 0.001 | 0.023 | 0.867 | 54 | 5.0 | 5.0 | 23.8 | 5.7 |
| 108-10-1 | TEXTURE LIKING | 0.001 | 0.023 | 0.869 | 54 | 1.5 | 2.6 | 23.8 | 5.7 |
| 15111-96-3 | TEXTURE LIKING | 0.000 | -0.022 | 0.875 | 54 | 1.2 | 1.2 | 23.8 | 5.7 |
| 623-42-7 | TEXTURE LIKING | 0.000 | 0.017 | 0.905 | 54 | 2780.2 | 1376.8 | 23.8 | 5.7 |
| 96-22-0 | TEXTURE LIKING | 0.000 | 0.015 | 0.915 | 54 | 51.2 | 18.6 | 23.8 | 5.7 |
| L* int | TEXTURE LIKING | 0.000 | -0.014 | 0.919 | 54 | 54.9 | 5.8 | 23.8 | 5.7 |
| 123-66-0 | TEXTURE LIKING | 0.000 | -0.007 | 0.957 | 54 | 108.2 | 128.0 | 23.8 | 5.7 |
| 1534-08-3 | TEXTURE LIKING | 0.000 | 0.002 | 0.988 | 54 | 0.4 | 0.2 | 23.8 | 5.7 |
| SSC | SWEETNESS INTENSITY | 0.690 | 0.831 | 0.000 | 54 | 7.4 | 1.4 | 23.0 | 4.8 |
| TOTAL SUGAR | SWEETNESS INTENSITY | 0.687 | 0.829 | 0.000 | 54 | 4473.9 | 1037.2 | 23.0 | 4.8 |
| SUCROSE | SWEETNESS INTENSITY | 0.445 | 0.667 | 0.000 | 54 | 1112.6 | 646.5 | 23.0 | 4.8 |
| 1629-58-9 | SWEETNESS INTENSITY | 0.377 | 0.614 | 0.000 | 54 | 117.9 | 65.9 | 23.0 | 4.8 |
| GLUCOSE | SWEETNESS INTENSITY | 0.338 | 0.581 | 0.000 | 54 | 1594.6 | 378.2 | 23.0 | 4.8 |
| FRUCTOSE | SWEETNESS INTENSITY | 0.300 | 0.548 | 0.000 | 54 | 1766.7 | 381.5 | 23.0 | 4.8 |
| 2639-63-6 | SWEETNESS INTENSITY | 0.296 | 0.544 | 0.000 | 54 | 10.8 | 13.0 | 23.0 | 4.8 |
| 2305-05-7 | SWEETNESS INTENSITY | 0.295 | 0.543 | 0.000 | 54 | 6.7 | 6.7 | 23.0 | 4.8 |
| 540-18-1 | SWEETNESS INTENSITY | 0.254 | 0.504 | 0.000 | 54 | 3.5 | 3.2 | 23.0 | 4.8 |
| 1576-87-0 | SWEETNESS INTENSITY | 0.242 | 0.492 | 0.000 | 54 | 37.5 | 20.1 | 23.0 | 4.8 |
| 142-92-7 | SWEETNESS INTENSITY | 0.239 | 0.489 | 0.000 | 54 | 53.5 | 49.7 | 23.0 | 4.8 |
| 60415-61-4 | SWEETNESS INTENSITY | 0.233 | 0.482 | 0.000 | 54 | 0.7 | 1.6 | 23.0 | 4.8 |
| 1576-86-9 | SWEETNESS INTENSITY | 0.217 | 0.466 | 0.000 | 54 | 37.8 | 21.7 | 23.0 | 4.8 |
| 109-21-7 | SWEETNESS INTENSITY | 0.198 | 0.445 | 0.001 | 54 | 72.1 | 157.2 | 23.0 | 4.8 |
| 111-71-7 | SWEETNESS INTENSITY | 0.195 | 0.441 | 0.001 | 54 | 3.2 | 2.3 | 23.0 | 4.8 |
| 3913-81-3 | SWEETNESS INTENSITY | 0.192 | 0.438 | 0.001 | 54 | 1.9 | 1.4 | 23.0 | 4.8 |
| L* int | SWEETNESS INTENSITY | 0.187 | 0.432 | 0.001 | 54 | 54.9 | 5.8 | 23.0 | 4.8 |
| 109-19-3 | SWEETNESS INTENSITY | 0.184 | 0.429 | 0.001 | 54 | 2.7 | 3.8 | 23.0 | 4.8 |
| 5989-33-3 | SWEETNESS INTENSITY | 0.171 | 0.414 | 0.002 | 54 | 2.8 | 2.4 | 23.0 | 4.8 |
| 123-86-4 | SWEETNESS INTENSITY | 0.158 | 0.397 | 0.003 | 54 | 73.5 | 85.0 | 23.0 | 4.8 |
| 706-14-9 | SWEETNESS INTENSITY | 0.151 | 0.388 | 0.004 | 54 | 44.5 | 81.1 | 23.0 | 4.8 |
| 638-11-9 | SWEETNESS INTENSITY | 0.151 | 0.388 | 0.004 | 54 | 72.7 | 67.2 | 23.0 | 4.8 |
| 110-93-0 | SWEETNESS INTENSITY | 0.151 | 0.388 | 0.004 | 54 | 2.7 | 1.5 | 23.0 | 4.8 |
| 591-78-6 | SWEETNESS INTENSITY | 0.144 | 0.379 | 0.005 | 54 | 10.3 | 13.9 | 23.0 | 4.8 |
| A* int | SWEETNESS INTENSITY | 0.139 | -0.373 | 0.005 | 54 | 28.8 | 7.6 | 23.0 | 4.8 |
| TOTAL VOLATILES | SWEETNESS INTENSITY | 0.139 | 0.373 | 0.005 | 54 | 15814.0 | 5238.5 | 23.0 | 4.8 |
| 124-19-6 | SWEETNESS INTENSITY | 0.139 | 0.372 | 0.006 | 54 | 8.5 | 7.5 | 23.0 | 4.8 |
| 5454-09-1 | SWEETNESS INTENSITY | 0.132 | 0.363 | 0.007 | 54 | 3.7 | 6.2 | 23.0 | 4.8 |
| CITRIC ACID | SWEETNESS INTENSITY | 0.124 | 0.353 | 0.009 | 54 | 741.0 | 147.2 | 23.0 | 4.8 |
| 110-39-4 | SWEETNESS INTENSITY | 0.123 | 0.351 | 0.009 | 54 | 40.8 | 70.0 | 23.0 | 4.8 |
| 53398-83-7 | SWEETNESS INTENSITY | 0.123 | 0.351 | 0.009 | 54 | 5.0 | 4.6 | 23.0 | 4.8 |
| 104-76-7 | SWEETNESS INTENSITY | 0.118 | 0.343 | 0.011 | 54 | 6.0 | 4.9 | 23.0 | 4.8 |
| 5881-17-4 | SWEETNESS INTENSITY | 0.112 | 0.334 | 0.014 | 54 | 6.2 | 2.5 | 23.0 | 4.8 |
| 4077-47-8 | SWEETNESS INTENSITY | 0.109 | 0.330 | 0.015 | 54 | 11.7 | 8.3 | 23.0 | 4.8 |
| 110-43-0 | SWEETNESS INTENSITY | 0.106 | 0.326 | 0.016 | 54 | 14.8 | 19.6 | 23.0 | 4.8 |
| TA | SWEETNESS INTENSITY | 0.094 | 0.307 | 0.024 | 54 | 0.8 | 0.1 | 23.0 | 4.8 |
| 616-25-1 | SWEETNESS INTENSITY | 0.094 | 0.307 | 0.024 | 54 | 15.9 | 7.3 | 23.0 | 4.8 |
| 10522-34-6 | SWEETNESS INTENSITY | 0.092 | 0.304 | 0.026 | 54 | 1.1 | 1.1 | 23.0 | 4.8 |
| 40716-66-3 | SWEETNESS INTENSITY | 0.092 | 0.303 | 0.026 | 54 | 84.3 | 107.5 | 23.0 | 4.8 |
| 623-42-7 | SWEETNESS INTENSITY | 0.085 | 0.292 | 0.032 | 54 | 2780.2 | 1376.8 | 23.0 | 4.8 |
| 2311-46-8 | SWEETNESS INTENSITY | 0.085 | 0.291 | 0.033 | 54 | 3.9 | 4.4 | 23.0 | 4.8 |
| 6728-26-3 | SWEETNESS INTENSITY | 0.078 | 0.278 | 0.041 | 54 | 8666.5 | 3359.7 | 23.0 | 4.8 |
| 105-66-8 | SWEETNESS INTENSITY | 0.077 | 0.278 | 0.042 | 54 | 5.0 | 3.7 | 23.0 | 4.8 |
| B* int | SWEETNESS INTENSITY | 0.075 | -0.275 | 0.044 | 54 | 25.8 | 4.5 | 23.0 | 4.8 |
| 4887-30-3 | SWEETNESS INTENSITY | 0.075 | 0.274 | 0.045 | 54 | 16.9 | 32.5 | 23.0 | 4.8 |
| 110-38-3 | SWEETNESS INTENSITY | 0.070 | 0.264 | 0.054 | 54 | 2.0 | 2.6 | 23.0 | 4.8 |
| 7786-58-5 | SWEETNESS INTENSITY | 0.069 | 0.262 | 0.056 | 54 | 12.2 | 28.4 | 23.0 | 4.8 |
| 112-14-1 | SWEETNESS INTENSITY | 0.066 | 0.257 | 0.061 | 54 | 18.3 | 24.0 | 23.0 | 4.8 |
| 2548-87-0 | SWEETNESS INTENSITY | 0.064 | 0.253 | 0.064 | 54 | 2.4 | 1.5 | 23.0 | 4.8 |
| 96-22-0 | SWEETNESS INTENSITY | 0.063 | 0.250 | 0.068 | 54 | 51.2 | 18.6 | 23.0 | 4.8 |
| 1191-16-8 | SWEETNESS INTENSITY | 0.063 | -0.250 | 0.068 | 54 | 5.5 | 7.0 | 23.0 | 4.8 |
| 105-54-4 | SWEETNESS INTENSITY | 0.062 | 0.249 | 0.069 | 54 | 42.0 | 17.1 | 23.0 | 4.8 |
| 124-13-0 | SWEETNESS INTENSITY | 0.053 | 0.231 | 0.093 | 54 | 5.9 | 3.0 | 23.0 | 4.8 |
| 29674-47-3 | SWEETNESS INTENSITY | 0.051 | 0.226 | 0.101 | 54 | 5.0 | 5.0 | 23.0 | 4.8 |
| pH | SWEETNESS INTENSITY | 0.048 | 0.220 | 0.110 | 54 | 3.7 | 0.2 | 23.0 | 4.8 |
| 71-41-0 | SWEETNESS INTENSITY | 0.045 | 0.213 | 0.122 | 54 | 1.0 | 1.3 | 23.0 | 4.8 |
| 628-63-7 | SWEETNESS INTENSITY | 0.040 | 0.199 | 0.149 | 54 | 4.6 | 1.7 | 23.0 | 4.8 |
| 556-24-1 | SWEETNESS INTENSITY | 0.039 | 0.198 | 0.151 | 54 | 46.6 | 57.0 | 23.0 | 4.8 |
| 564-94-3 | SWEETNESS INTENSITY | 0.030 | 0.173 | 0.211 | 54 | 6.5 | 7.4 | 23.0 | 4.8 |
| 20664-46-4 | SWEETNESS INTENSITY | 0.029 | 0.171 | 0.215 | 54 | 20.6 | 19.0 | 23.0 | 4.8 |
| 624-41-9 | SWEETNESS INTENSITY | 0.026 | -0.162 | 0.243 | 54 | 18.9 | 18.1 | 23.0 | 4.8 |
| 539-82-2 | SWEETNESS INTENSITY | 0.025 | 0.158 | 0.255 | 54 | 3.3 | 3.9 | 23.0 | 4.8 |
| 1534-08-3 | SWEETNESS INTENSITY | 0.021 | 0.146 | 0.292 | 54 | 0.4 | 0.2 | 23.0 | 4.8 |
| 106-70-7 | SWEETNESS INTENSITY | 0.020 | 0.142 | 0.307 | 54 | 252.7 | 164.0 | 23.0 | 4.8 |
| B* ext | SWEETNESS INTENSITY | 0.019 | -0.137 | 0.324 | 54 | 19.0 | 3.3 | 23.0 | 4.8 |
| 116-53-0 | SWEETNESS INTENSITY | 0.018 | 0.133 | 0.339 | 54 | 19.7 | 16.0 | 23.0 | 4.8 |
| A* ext | SWEETNESS INTENSITY | 0.017 | 0.130 | 0.348 | 54 | 36.4 | 3.1 | 23.0 | 4.8 |
| 78-70-6 | SWEETNESS INTENSITY | 0.017 | 0.130 | 0.350 | 54 | 128.8 | 113.0 | 23.0 | 4.8 |
| 123-66-0 | SWEETNESS INTENSITY | 0.017 | 0.129 | 0.352 | 54 | 108.2 | 128.0 | 23.0 | 4.8 |
| 55514-48-2 | SWEETNESS INTENSITY | 0.014 | -0.120 | 0.387 | 54 | 0.5 | 0.5 | 23.0 | 4.8 |
| 7452-79-1 | SWEETNESS INTENSITY | 0.013 | 0.116 | 0.405 | 54 | 50.0 | 31.0 | 23.0 | 4.8 |
| 821-55-6 | SWEETNESS INTENSITY | 0.013 | 0.113 | 0.416 | 54 | 3.5 | 8.2 | 23.0 | 4.8 |
| 66-25-1 | SWEETNESS INTENSITY | 0.011 | 0.104 | 0.455 | 54 | 2545.9 | 1722.0 | 23.0 | 4.8 |
| 928-95-0 | SWEETNESS INTENSITY | 0.010 | -0.101 | 0.468 | 54 | 66.8 | 61.7 | 23.0 | 4.8 |
| 589-38-8 | SWEETNESS INTENSITY | 0.009 | 0.097 | 0.484 | 54 | 1.9 | 1.2 | 23.0 | 4.8 |
| 110-62-3 | SWEETNESS INTENSITY | 0.009 | -0.095 | 0.496 | 54 | 7.9 | 8.9 | 23.0 | 4.8 |
| 109-60-4 | SWEETNESS INTENSITY | 0.009 | -0.095 | 0.496 | 54 | 3.8 | 2.7 | 23.0 | 4.8 |
| 111-27-3 | SWEETNESS INTENSITY | 0.009 | -0.094 | 0.501 | 54 | 45.5 | 94.6 | 23.0 | 4.8 |
| 140-11-4 | SWEETNESS INTENSITY | 0.009 | -0.093 | 0.501 | 54 | 11.1 | 8.6 | 23.0 | 4.8 |
| MALIC ACID | SWEETNESS INTENSITY | 0.009 | -0.093 | 0.506 | 54 | 212.4 | 51.6 | 23.0 | 4.8 |
| FORCE | SWEETNESS INTENSITY | 0.005 | -0.074 | 0.594 | 54 | 0.6 | 0.2 | 23.0 | 4.8 |
| 128-37-0 | SWEETNESS INTENSITY | 0.004 | 0.061 | 0.664 | 54 | 4.1 | 3.7 | 23.0 | 4.8 |
| 106-32-1 | SWEETNESS INTENSITY | 0.003 | -0.055 | 0.694 | 54 | 2.2 | 3.0 | 23.0 | 4.8 |
| 623-43-8 | SWEETNESS INTENSITY | 0.003 | -0.050 | 0.718 | 54 | 3.4 | 3.4 | 23.0 | 4.8 |
| 108-10-1 | SWEETNESS INTENSITY | 0.002 | -0.050 | 0.721 | 54 | 1.5 | 2.6 | 23.0 | 4.8 |
| 29811-50-5 | SWEETNESS INTENSITY | 0.002 | 0.048 | 0.728 | 54 | 3.2 | 5.3 | 23.0 | 4.8 |
| 15111-96-3 | SWEETNESS INTENSITY | 0.002 | 0.048 | 0.731 | 54 | 1.2 | 1.2 | 23.0 | 4.8 |
| 2432-51-1 | SWEETNESS INTENSITY | 0.002 | 0.041 | 0.770 | 54 | 4.4 | 5.8 | 23.0 | 4.8 |
| 624-24-8 | SWEETNESS INTENSITY | 0.002 | 0.039 | 0.779 | 54 | 5.6 | 3.7 | 23.0 | 4.8 |
| 134-20-3 | SWEETNESS INTENSITY | 0.001 | 0.030 | 0.827 | 54 | 0.1 | 0.7 | 23.0 | 4.8 |
| 1576-95-0 | SWEETNESS INTENSITY | 0.000 | 0.018 | 0.899 | 54 | 2.1 | 2.0 | 23.0 | 4.8 |
| 123-92-2 | SWEETNESS INTENSITY | 0.000 | -0.017 | 0.905 | 54 | 23.0 | 21.5 | 23.0 | 4.8 |
| 2497-18-9 | SWEETNESS INTENSITY | 0.000 | -0.012 | 0.934 | 54 | 24.9 | 21.5 | 23.0 | 4.8 |
| L* ext | SWEETNESS INTENSITY | 0.000 | 0.010 | 0.943 | 54 | 33.6 | 2.6 | 23.0 | 4.8 |
| 103-09-3 | SWEETNESS INTENSITY | 0.000 | -0.007 | 0.960 | 54 | 3.0 | 1.1 | 23.0 | 4.8 |
| 105-37-3 | SWEETNESS INTENSITY | 0.000 | 0.005 | 0.973 | 54 | 10.1 | 14.1 | 23.0 | 4.8 |
| 75-85-4 | SWEETNESS INTENSITY | 0.000 | -0.001 | 0.995 | 54 | 3.9 | 2.2 | 23.0 | 4.8 |
| 96-04-8 | SWEETNESS INTENSITY | 0.000 | -0.001 | 0.996 | 54 | 3.1 | 8.0 | 23.0 | 4.8 |
| TA | SOURNESS INTENSITY | 0.314 | 0.561 | 0.000 | 54 | 0.8 | 0.1 | 18.1 | 3.1 |
| MALIC ACID | SOURNESS INTENSITY | 0.189 | 0.435 | 0.001 | 54 | 212.4 | 51.6 | 18.1 | 3.1 |
| CITRIC ACID | SOURNESS INTENSITY | 0.146 | 0.382 | 0.004 | 54 | 741.0 | 147.2 | 18.1 | 3.1 |
| 134-20-3 | SOURNESS INTENSITY | 0.137 | -0.370 | 0.006 | 54 | 0.1 | 0.7 | 18.1 | 3.1 |
| pH | SOURNESS INTENSITY | 0.118 | -0.344 | 0.011 | 54 | 3.7 | 0.2 | 18.1 | 3.1 |
| 15111-96-3 | SOURNESS INTENSITY | 0.106 | -0.325 | 0.016 | 54 | 1.2 | 1.2 | 18.1 | 3.1 |
| 624-41-9 | SOURNESS INTENSITY | 0.097 | 0.311 | 0.022 | 54 | 18.9 | 18.1 | 18.1 | 3.1 |
| FRUCTOSE | SOURNESS INTENSITY | 0.089 | -0.298 | 0.029 | 54 | 1766.7 | 381.5 | 18.1 | 3.1 |
| GLUCOSE | SOURNESS INTENSITY | 0.075 | -0.274 | 0.045 | 54 | 1594.6 | 378.2 | 18.1 | 3.1 |
| 4887-30-3 | SOURNESS INTENSITY | 0.073 | -0.270 | 0.048 | 54 | 16.9 | 32.5 | 18.1 | 3.1 |
| 1191-16-8 | SOURNESS INTENSITY | 0.068 | 0.261 | 0.056 | 54 | 5.5 | 7.0 | 18.1 | 3.1 |
| 78-70-6 | SOURNESS INTENSITY | 0.068 | 0.260 | 0.058 | 54 | 128.8 | 113.0 | 18.1 | 3.1 |
| 5454-09-1 | SOURNESS INTENSITY | 0.067 | -0.259 | 0.058 | 54 | 3.7 | 6.2 | 18.1 | 3.1 |
| 110-39-4 | SOURNESS INTENSITY | 0.066 | -0.257 | 0.061 | 54 | 40.8 | 70.0 | 18.1 | 3.1 |
| 589-38-8 | SOURNESS INTENSITY | 0.062 | 0.248 | 0.070 | 54 | 1.9 | 1.2 | 18.1 | 3.1 |
| 624-24-8 | SOURNESS INTENSITY | 0.054 | -0.233 | 0.090 | 54 | 5.6 | 3.7 | 18.1 | 3.1 |
| 928-95-0 | SOURNESS INTENSITY | 0.050 | 0.223 | 0.106 | 54 | 66.8 | 61.7 | 18.1 | 3.1 |
| 128-37-0 | SOURNESS INTENSITY | 0.049 | 0.221 | 0.108 | 54 | 4.1 | 3.7 | 18.1 | 3.1 |
| 110-62-3 | SOURNESS INTENSITY | 0.047 | -0.218 | 0.113 | 54 | 7.9 | 8.9 | 18.1 | 3.1 |
| 2497-18-9 | SOURNESS INTENSITY | 0.036 | 0.189 | 0.171 | 54 | 24.9 | 21.5 | 18.1 | 3.1 |
| 111-27-3 | SOURNESS INTENSITY | 0.036 | 0.189 | 0.172 | 54 | 45.5 | 94.6 | 18.1 | 3.1 |
| 96-04-8 | SOURNESS INTENSITY | 0.033 | 0.182 | 0.188 | 54 | 3.1 | 8.0 | 18.1 | 3.1 |
| 104-76-7 | SOURNESS INTENSITY | 0.032 | -0.178 | 0.198 | 54 | 6.0 | 4.9 | 18.1 | 3.1 |
| 10522-34-6 | SOURNESS INTENSITY | 0.031 | -0.177 | 0.201 | 54 | 1.1 | 1.1 | 18.1 | 3.1 |
| 140-11-4 | SOURNESS INTENSITY | 0.031 | 0.176 | 0.203 | 54 | 11.1 | 8.6 | 18.1 | 3.1 |
| L* ext | SOURNESS INTENSITY | 0.030 | 0.172 | 0.212 | 54 | 33.6 | 2.6 | 18.1 | 3.1 |
| 556-24-1 | SOURNESS INTENSITY | 0.028 | -0.167 | 0.226 | 54 | 46.6 | 57.0 | 18.1 | 3.1 |
| 55514-48-2 | SOURNESS INTENSITY | 0.027 | 0.165 | 0.232 | 54 | 0.5 | 0.5 | 18.1 | 3.1 |
| 2548-87-0 | SOURNESS INTENSITY | 0.027 | 0.165 | 0.233 | 54 | 2.4 | 1.5 | 18.1 | 3.1 |
| 623-43-8 | SOURNESS INTENSITY | 0.024 | -0.154 | 0.267 | 54 | 3.4 | 3.4 | 18.1 | 3.1 |
| TOTAL SUGAR | SOURNESS INTENSITY | 0.022 | -0.148 | 0.287 | 54 | 4473.9 | 1037.2 | 18.1 | 3.1 |
| L* int | SOURNESS INTENSITY | 0.021 | -0.145 | 0.296 | 54 | 54.9 | 5.8 | 18.1 | 3.1 |
| SSC | SOURNESS INTENSITY | 0.020 | -0.142 | 0.305 | 54 | 7.4 | 1.4 | 18.1 | 3.1 |
| 112-14-1 | SOURNESS INTENSITY | 0.020 | -0.141 | 0.311 | 54 | 18.3 | 24.0 | 18.1 | 3.1 |
| B* ext | SOURNESS INTENSITY | 0.020 | 0.140 | 0.312 | 54 | 19.0 | 3.3 | 18.1 | 3.1 |
| B* int | SOURNESS INTENSITY | 0.020 | 0.140 | 0.314 | 54 | 25.8 | 4.5 | 18.1 | 3.1 |
| 103-09-3 | SOURNESS INTENSITY | 0.017 | 0.129 | 0.354 | 54 | 3.0 | 1.1 | 18.1 | 3.1 |
| 1534-08-3 | SOURNESS INTENSITY | 0.016 | -0.127 | 0.359 | 54 | 0.4 | 0.2 | 18.1 | 3.1 |
| 106-70-7 | SOURNESS INTENSITY | 0.016 | -0.125 | 0.366 | 54 | 252.7 | 164.0 | 18.1 | 3.1 |
| 110-43-0 | SOURNESS INTENSITY | 0.015 | -0.121 | 0.381 | 54 | 14.8 | 19.6 | 18.1 | 3.1 |
| 53398-83-7 | SOURNESS INTENSITY | 0.014 | -0.120 | 0.389 | 54 | 5.0 | 4.6 | 18.1 | 3.1 |
| 5989-33-3 | SOURNESS INTENSITY | 0.014 | 0.117 | 0.398 | 54 | 2.8 | 2.4 | 18.1 | 3.1 |
| 5881-17-4 | SOURNESS INTENSITY | 0.014 | 0.117 | 0.401 | 54 | 6.2 | 2.5 | 18.1 | 3.1 |
| 7452-79-1 | SOURNESS INTENSITY | 0.013 | -0.114 | 0.411 | 54 | 50.0 | 31.0 | 18.1 | 3.1 |
| 1576-95-0 | SOURNESS INTENSITY | 0.013 | 0.114 | 0.413 | 54 | 2.1 | 2.0 | 18.1 | 3.1 |
| 20664-46-4 | SOURNESS INTENSITY | 0.012 | 0.111 | 0.423 | 54 | 20.6 | 19.0 | 18.1 | 3.1 |
| 29811-50-5 | SOURNESS INTENSITY | 0.012 | 0.108 | 0.436 | 54 | 3.2 | 5.3 | 18.1 | 3.1 |
| 110-38-3 | SOURNESS INTENSITY | 0.012 | -0.108 | 0.437 | 54 | 2.0 | 2.6 | 18.1 | 3.1 |
| 2639-63-6 | SOURNESS INTENSITY | 0.011 | -0.107 | 0.443 | 54 | 10.8 | 13.0 | 18.1 | 3.1 |
| 123-92-2 | SOURNESS INTENSITY | 0.011 | 0.105 | 0.450 | 54 | 23.0 | 21.5 | 18.1 | 3.1 |
| 109-21-7 | SOURNESS INTENSITY | 0.011 | -0.105 | 0.451 | 54 | 72.1 | 157.2 | 18.1 | 3.1 |
| 110-93-0 | SOURNESS INTENSITY | 0.011 | 0.105 | 0.452 | 54 | 2.7 | 1.5 | 18.1 | 3.1 |
| 29674-47-3 | SOURNESS INTENSITY | 0.011 | 0.104 | 0.454 | 54 | 5.0 | 5.0 | 18.1 | 3.1 |
| SUCROSE | SOURNESS INTENSITY | 0.010 | 0.099 | 0.476 | 54 | 1112.6 | 646.5 | 18.1 | 3.1 |
| 4077-47-8 | SOURNESS INTENSITY | 0.009 | 0.092 | 0.507 | 54 | 11.7 | 8.3 | 18.1 | 3.1 |
| 539-82-2 | SOURNESS INTENSITY | 0.008 | 0.092 | 0.510 | 54 | 3.3 | 3.9 | 18.1 | 3.1 |
| 591-78-6 | SOURNESS INTENSITY | 0.008 | 0.091 | 0.514 | 54 | 10.3 | 13.9 | 18.1 | 3.1 |
| A* int | SOURNESS INTENSITY | 0.008 | 0.088 | 0.525 | 54 | 28.8 | 7.6 | 18.1 | 3.1 |
| 109-19-3 | SOURNESS INTENSITY | 0.007 | -0.086 | 0.537 | 54 | 2.7 | 3.8 | 18.1 | 3.1 |
| 60415-61-4 | SOURNESS INTENSITY | 0.007 | -0.084 | 0.545 | 54 | 0.7 | 1.6 | 18.1 | 3.1 |
| 142-92-7 | SOURNESS INTENSITY | 0.007 | -0.081 | 0.559 | 54 | 53.5 | 49.7 | 18.1 | 3.1 |
| 111-71-7 | SOURNESS INTENSITY | 0.006 | 0.075 | 0.589 | 54 | 3.2 | 2.3 | 18.1 | 3.1 |
| 106-32-1 | SOURNESS INTENSITY | 0.006 | 0.075 | 0.591 | 54 | 2.2 | 3.0 | 18.1 | 3.1 |
| 66-25-1 | SOURNESS INTENSITY | 0.005 | 0.072 | 0.605 | 54 | 2545.9 | 1722.0 | 18.1 | 3.1 |
| 564-94-3 | SOURNESS INTENSITY | 0.005 | 0.072 | 0.606 | 54 | 6.5 | 7.4 | 18.1 | 3.1 |
| 116-53-0 | SOURNESS INTENSITY | 0.005 | -0.068 | 0.625 | 54 | 19.7 | 16.0 | 18.1 | 3.1 |
| 109-60-4 | SOURNESS INTENSITY | 0.005 | 0.068 | 0.626 | 54 | 3.8 | 2.7 | 18.1 | 3.1 |
| 2432-51-1 | SOURNESS INTENSITY | 0.004 | -0.064 | 0.644 | 54 | 4.4 | 5.8 | 18.1 | 3.1 |
| 1576-86-9 | SOURNESS INTENSITY | 0.004 | 0.064 | 0.645 | 54 | 37.8 | 21.7 | 18.1 | 3.1 |
| 124-13-0 | SOURNESS INTENSITY | 0.004 | 0.061 | 0.660 | 54 | 5.9 | 3.0 | 18.1 | 3.1 |
| 3913-81-3 | SOURNESS INTENSITY | 0.004 | -0.060 | 0.669 | 54 | 1.9 | 1.4 | 18.1 | 3.1 |
| 1629-58-9 | SOURNESS INTENSITY | 0.003 | 0.058 | 0.678 | 54 | 117.9 | 65.9 | 18.1 | 3.1 |
| 628-63-7 | SOURNESS INTENSITY | 0.003 | -0.052 | 0.709 | 54 | 4.6 | 1.7 | 18.1 | 3.1 |
| 7786-58-5 | SOURNESS INTENSITY | 0.003 | -0.051 | 0.713 | 54 | 12.2 | 28.4 | 18.1 | 3.1 |
| 96-22-0 | SOURNESS INTENSITY | 0.002 | 0.050 | 0.721 | 54 | 51.2 | 18.6 | 18.1 | 3.1 |
| 616-25-1 | SOURNESS INTENSITY | 0.002 | 0.048 | 0.731 | 54 | 15.9 | 7.3 | 18.1 | 3.1 |
| 75-85-4 | SOURNESS INTENSITY | 0.002 | 0.047 | 0.737 | 54 | 3.9 | 2.2 | 18.1 | 3.1 |
| 706-14-9 | SOURNESS INTENSITY | 0.002 | -0.040 | 0.772 | 54 | 44.5 | 81.1 | 18.1 | 3.1 |
| A* ext | SOURNESS INTENSITY | 0.002 | -0.040 | 0.774 | 54 | 36.4 | 3.1 | 18.1 | 3.1 |
| 2311-46-8 | SOURNESS INTENSITY | 0.002 | 0.039 | 0.779 | 54 | 3.9 | 4.4 | 18.1 | 3.1 |
| TOTAL VOLATILES | SOURNESS INTENSITY | 0.001 | 0.029 | 0.834 | 54 | 15814.0 | 5238.5 | 18.1 | 3.1 |
| 40716-66-3 | SOURNESS INTENSITY | 0.001 | -0.029 | 0.838 | 54 | 84.3 | 107.5 | 18.1 | 3.1 |
| 123-66-0 | SOURNESS INTENSITY | 0.001 | -0.027 | 0.849 | 54 | 108.2 | 128.0 | 18.1 | 3.1 |
| 1576-87-0 | SOURNESS INTENSITY | 0.001 | 0.026 | 0.851 | 54 | 37.5 | 20.1 | 18.1 | 3.1 |
| 71-41-0 | SOURNESS INTENSITY | 0.001 | -0.025 | 0.855 | 54 | 1.0 | 1.3 | 18.1 | 3.1 |
| 124-19-6 | SOURNESS INTENSITY | 0.000 | 0.021 | 0.883 | 54 | 8.5 | 7.5 | 18.1 | 3.1 |
| 108-10-1 | SOURNESS INTENSITY | 0.000 | 0.020 | 0.886 | 54 | 1.5 | 2.6 | 18.1 | 3.1 |
| 638-11-9 | SOURNESS INTENSITY | 0.000 | -0.018 | 0.899 | 54 | 72.7 | 67.2 | 18.1 | 3.1 |
| 6728-26-3 | SOURNESS INTENSITY | 0.000 | 0.016 | 0.909 | 54 | 8666.5 | 3359.7 | 18.1 | 3.1 |
| 123-86-4 | SOURNESS INTENSITY | 0.000 | -0.016 | 0.910 | 54 | 73.5 | 85.0 | 18.1 | 3.1 |
| 105-66-8 | SOURNESS INTENSITY | 0.000 | 0.011 | 0.938 | 54 | 5.0 | 3.7 | 18.1 | 3.1 |
| 105-54-4 | SOURNESS INTENSITY | 0.000 | -0.011 | 0.939 | 54 | 42.0 | 17.1 | 18.1 | 3.1 |
| FORCE | SOURNESS INTENSITY | 0.000 | 0.008 | 0.953 | 54 | 0.6 | 0.2 | 18.1 | 3.1 |
| 540-18-1 | SOURNESS INTENSITY | 0.000 | -0.008 | 0.957 | 54 | 3.5 | 3.2 | 18.1 | 3.1 |
| 2305-05-7 | SOURNESS INTENSITY | 0.000 | -0.007 | 0.962 | 54 | 6.7 | 6.7 | 18.1 | 3.1 |
| 105-37-3 | SOURNESS INTENSITY | 0.000 | -0.006 | 0.964 | 54 | 10.1 | 14.1 | 18.1 | 3.1 |
| 623-42-7 | SOURNESS INTENSITY | 0.000 | -0.006 | 0.966 | 54 | 2780.2 | 1376.8 | 18.1 | 3.1 |
| 821-55-6 | SOURNESS INTENSITY | 0.000 | 0.001 | 0.997 | 54 | 3.5 | 8.2 | 18.1 | 3.1 |
| SSC | STRAWBERRY FLAVOR INTENSITY | 0.584 | 0.764 | 0.000 | 54 | 7.4 | 1.4 | 26.9 | 3.9 |
| TOTAL SUGAR | STRAWBERRY FLAVOR INTENSITY | 0.569 | 0.755 | 0.000 | 54 | 4473.9 | 1037.2 | 26.9 | 3.9 |
| SUCROSE | STRAWBERRY FLAVOR INTENSITY | 0.498 | 0.705 | 0.000 | 54 | 1112.6 | 646.5 | 26.9 | 3.9 |
| 1629-58-9 | STRAWBERRY FLAVOR INTENSITY | 0.356 | 0.597 | 0.000 | 54 | 117.9 | 65.9 | 26.9 | 3.9 |
| 2305-05-7 | STRAWBERRY FLAVOR INTENSITY | 0.283 | 0.532 | 0.000 | 54 | 6.7 | 6.7 | 26.9 | 3.9 |
| 540-18-1 | STRAWBERRY FLAVOR INTENSITY | 0.260 | 0.509 | 0.000 | 54 | 3.5 | 3.2 | 26.9 | 3.9 |
| TA | STRAWBERRY FLAVOR INTENSITY | 0.256 | 0.506 | 0.000 | 54 | 0.8 | 0.1 | 26.9 | 3.9 |
| 1576-87-0 | STRAWBERRY FLAVOR INTENSITY | 0.239 | 0.488 | 0.000 | 54 | 37.5 | 20.1 | 26.9 | 3.9 |
| 2639-63-6 | STRAWBERRY FLAVOR INTENSITY | 0.235 | 0.485 | 0.000 | 54 | 10.8 | 13.0 | 26.9 | 3.9 |
| CITRIC ACID | STRAWBERRY FLAVOR INTENSITY | 0.235 | 0.485 | 0.000 | 54 | 741.0 | 147.2 | 26.9 | 3.9 |
| 60415-61-4 | STRAWBERRY FLAVOR INTENSITY | 0.233 | 0.482 | 0.000 | 54 | 0.7 | 1.6 | 26.9 | 3.9 |
| 142-92-7 | STRAWBERRY FLAVOR INTENSITY | 0.227 | 0.477 | 0.000 | 54 | 53.5 | 49.7 | 26.9 | 3.9 |
| 1576-86-9 | STRAWBERRY FLAVOR INTENSITY | 0.222 | 0.472 | 0.000 | 54 | 37.8 | 21.7 | 26.9 | 3.9 |
| 5989-33-3 | STRAWBERRY FLAVOR INTENSITY | 0.208 | 0.456 | 0.001 | 54 | 2.8 | 2.4 | 26.9 | 3.9 |
| GLUCOSE | STRAWBERRY FLAVOR INTENSITY | 0.205 | 0.453 | 0.001 | 54 | 1594.6 | 378.2 | 26.9 | 3.9 |
| 123-86-4 | STRAWBERRY FLAVOR INTENSITY | 0.205 | 0.453 | 0.001 | 54 | 73.5 | 85.0 | 26.9 | 3.9 |
| 111-71-7 | STRAWBERRY FLAVOR INTENSITY | 0.201 | 0.448 | 0.001 | 54 | 3.2 | 2.3 | 26.9 | 3.9 |
| 109-21-7 | STRAWBERRY FLAVOR INTENSITY | 0.194 | 0.440 | 0.001 | 54 | 72.1 | 157.2 | 26.9 | 3.9 |
| 591-78-6 | STRAWBERRY FLAVOR INTENSITY | 0.180 | 0.425 | 0.001 | 54 | 10.3 | 13.9 | 26.9 | 3.9 |
| 109-19-3 | STRAWBERRY FLAVOR INTENSITY | 0.179 | 0.424 | 0.001 | 54 | 2.7 | 3.8 | 26.9 | 3.9 |
| 706-14-9 | STRAWBERRY FLAVOR INTENSITY | 0.179 | 0.423 | 0.001 | 54 | 44.5 | 81.1 | 26.9 | 3.9 |
| TOTAL VOLATILES | STRAWBERRY FLAVOR INTENSITY | 0.167 | 0.409 | 0.002 | 54 | 15814.0 | 5238.5 | 26.9 | 3.9 |
| FRUCTOSE | STRAWBERRY FLAVOR INTENSITY | 0.166 | 0.407 | 0.002 | 54 | 1766.7 | 381.5 | 26.9 | 3.9 |
| 638-11-9 | STRAWBERRY FLAVOR INTENSITY | 0.164 | 0.405 | 0.002 | 54 | 72.7 | 67.2 | 26.9 | 3.9 |
| 3913-81-3 | STRAWBERRY FLAVOR INTENSITY | 0.158 | 0.398 | 0.003 | 54 | 1.9 | 1.4 | 26.9 | 3.9 |
| 616-25-1 | STRAWBERRY FLAVOR INTENSITY | 0.154 | 0.392 | 0.003 | 54 | 15.9 | 7.3 | 26.9 | 3.9 |
| 5881-17-4 | STRAWBERRY FLAVOR INTENSITY | 0.153 | 0.391 | 0.003 | 54 | 6.2 | 2.5 | 26.9 | 3.9 |
| 110-93-0 | STRAWBERRY FLAVOR INTENSITY | 0.137 | 0.370 | 0.006 | 54 | 2.7 | 1.5 | 26.9 | 3.9 |
| 124-19-6 | STRAWBERRY FLAVOR INTENSITY | 0.129 | 0.359 | 0.008 | 54 | 8.5 | 7.5 | 26.9 | 3.9 |
| 40716-66-3 | STRAWBERRY FLAVOR INTENSITY | 0.112 | 0.335 | 0.013 | 54 | 84.3 | 107.5 | 26.9 | 3.9 |
| L* int | STRAWBERRY FLAVOR INTENSITY | 0.109 | 0.330 | 0.015 | 54 | 54.9 | 5.8 | 26.9 | 3.9 |
| 4077-47-8 | STRAWBERRY FLAVOR INTENSITY | 0.108 | 0.328 | 0.015 | 54 | 11.7 | 8.3 | 26.9 | 3.9 |
| 2311-46-8 | STRAWBERRY FLAVOR INTENSITY | 0.103 | 0.322 | 0.018 | 54 | 3.9 | 4.4 | 26.9 | 3.9 |
| 110-43-0 | STRAWBERRY FLAVOR INTENSITY | 0.101 | 0.318 | 0.019 | 54 | 14.8 | 19.6 | 26.9 | 3.9 |
| 623-42-7 | STRAWBERRY FLAVOR INTENSITY | 0.097 | 0.312 | 0.022 | 54 | 2780.2 | 1376.8 | 26.9 | 3.9 |
| 6728-26-3 | STRAWBERRY FLAVOR INTENSITY | 0.096 | 0.310 | 0.022 | 54 | 8666.5 | 3359.7 | 26.9 | 3.9 |
| 105-54-4 | STRAWBERRY FLAVOR INTENSITY | 0.089 | 0.299 | 0.028 | 54 | 42.0 | 17.1 | 26.9 | 3.9 |
| A* int | STRAWBERRY FLAVOR INTENSITY | 0.087 | -0.295 | 0.030 | 54 | 28.8 | 7.6 | 26.9 | 3.9 |
| 110-62-3 | STRAWBERRY FLAVOR INTENSITY | 0.079 | -0.281 | 0.039 | 54 | 7.9 | 8.9 | 26.9 | 3.9 |
| 110-39-4 | STRAWBERRY FLAVOR INTENSITY | 0.079 | 0.281 | 0.040 | 54 | 40.8 | 70.0 | 26.9 | 3.9 |
| 78-70-6 | STRAWBERRY FLAVOR INTENSITY | 0.074 | 0.272 | 0.046 | 54 | 128.8 | 113.0 | 26.9 | 3.9 |
| 29674-47-3 | STRAWBERRY FLAVOR INTENSITY | 0.069 | 0.263 | 0.055 | 54 | 5.0 | 5.0 | 26.9 | 3.9 |
| 5454-09-1 | STRAWBERRY FLAVOR INTENSITY | 0.067 | 0.259 | 0.059 | 54 | 3.7 | 6.2 | 26.9 | 3.9 |
| 96-22-0 | STRAWBERRY FLAVOR INTENSITY | 0.066 | 0.258 | 0.060 | 54 | 51.2 | 18.6 | 26.9 | 3.9 |
| 104-76-7 | STRAWBERRY FLAVOR INTENSITY | 0.064 | 0.252 | 0.066 | 54 | 6.0 | 4.9 | 26.9 | 3.9 |
| 10522-34-6 | STRAWBERRY FLAVOR INTENSITY | 0.060 | 0.246 | 0.073 | 54 | 1.1 | 1.1 | 26.9 | 3.9 |
| 7786-58-5 | STRAWBERRY FLAVOR INTENSITY | 0.059 | 0.243 | 0.077 | 54 | 12.2 | 28.4 | 26.9 | 3.9 |
| 124-13-0 | STRAWBERRY FLAVOR INTENSITY | 0.057 | 0.239 | 0.081 | 54 | 5.9 | 3.0 | 26.9 | 3.9 |
| 112-14-1 | STRAWBERRY FLAVOR INTENSITY | 0.056 | 0.237 | 0.084 | 54 | 18.3 | 24.0 | 26.9 | 3.9 |
| 2548-87-0 | STRAWBERRY FLAVOR INTENSITY | 0.056 | 0.236 | 0.086 | 54 | 2.4 | 1.5 | 26.9 | 3.9 |
| 628-63-7 | STRAWBERRY FLAVOR INTENSITY | 0.042 | 0.206 | 0.135 | 54 | 4.6 | 1.7 | 26.9 | 3.9 |
| B* int | STRAWBERRY FLAVOR INTENSITY | 0.039 | -0.198 | 0.152 | 54 | 25.8 | 4.5 | 26.9 | 3.9 |
| 105-66-8 | STRAWBERRY FLAVOR INTENSITY | 0.037 | 0.193 | 0.162 | 54 | 5.0 | 3.7 | 26.9 | 3.9 |
| 20664-46-4 | STRAWBERRY FLAVOR INTENSITY | 0.037 | 0.193 | 0.162 | 54 | 20.6 | 19.0 | 26.9 | 3.9 |
| 564-94-3 | STRAWBERRY FLAVOR INTENSITY | 0.037 | 0.192 | 0.165 | 54 | 6.5 | 7.4 | 26.9 | 3.9 |
| 4887-30-3 | STRAWBERRY FLAVOR INTENSITY | 0.034 | 0.185 | 0.180 | 54 | 16.9 | 32.5 | 26.9 | 3.9 |
| 110-38-3 | STRAWBERRY FLAVOR INTENSITY | 0.031 | 0.176 | 0.204 | 54 | 2.0 | 2.6 | 26.9 | 3.9 |
| 53398-83-7 | STRAWBERRY FLAVOR INTENSITY | 0.029 | 0.172 | 0.215 | 54 | 5.0 | 4.6 | 26.9 | 3.9 |
| 539-82-2 | STRAWBERRY FLAVOR INTENSITY | 0.026 | 0.160 | 0.247 | 54 | 3.3 | 3.9 | 26.9 | 3.9 |
| 134-20-3 | STRAWBERRY FLAVOR INTENSITY | 0.021 | -0.145 | 0.295 | 54 | 0.1 | 0.7 | 26.9 | 3.9 |
| 29811-50-5 | STRAWBERRY FLAVOR INTENSITY | 0.020 | 0.143 | 0.303 | 54 | 3.2 | 5.3 | 26.9 | 3.9 |
| 556-24-1 | STRAWBERRY FLAVOR INTENSITY | 0.019 | 0.137 | 0.324 | 54 | 46.6 | 57.0 | 26.9 | 3.9 |
| 589-38-8 | STRAWBERRY FLAVOR INTENSITY | 0.018 | 0.136 | 0.328 | 54 | 1.9 | 1.2 | 26.9 | 3.9 |
| 821-55-6 | STRAWBERRY FLAVOR INTENSITY | 0.017 | 0.130 | 0.348 | 54 | 3.5 | 8.2 | 26.9 | 3.9 |
| 2432-51-1 | STRAWBERRY FLAVOR INTENSITY | 0.017 | -0.129 | 0.351 | 54 | 4.4 | 5.8 | 26.9 | 3.9 |
| 7452-79-1 | STRAWBERRY FLAVOR INTENSITY | 0.016 | 0.125 | 0.369 | 54 | 50.0 | 31.0 | 26.9 | 3.9 |
| 66-25-1 | STRAWBERRY FLAVOR INTENSITY | 0.016 | 0.125 | 0.369 | 54 | 2545.9 | 1722.0 | 26.9 | 3.9 |
| 116-53-0 | STRAWBERRY FLAVOR INTENSITY | 0.015 | 0.122 | 0.379 | 54 | 19.7 | 16.0 | 26.9 | 3.9 |
| 128-37-0 | STRAWBERRY FLAVOR INTENSITY | 0.013 | 0.115 | 0.407 | 54 | 4.1 | 3.7 | 26.9 | 3.9 |
| pH | STRAWBERRY FLAVOR INTENSITY | 0.010 | 0.102 | 0.464 | 54 | 3.7 | 0.2 | 26.9 | 3.9 |
| B* ext | STRAWBERRY FLAVOR INTENSITY | 0.009 | -0.097 | 0.488 | 54 | 19.0 | 3.3 | 26.9 | 3.9 |
| 106-70-7 | STRAWBERRY FLAVOR INTENSITY | 0.009 | 0.096 | 0.491 | 54 | 252.7 | 164.0 | 26.9 | 3.9 |
| 624-24-8 | STRAWBERRY FLAVOR INTENSITY | 0.009 | -0.094 | 0.498 | 54 | 5.6 | 3.7 | 26.9 | 3.9 |
| 623-43-8 | STRAWBERRY FLAVOR INTENSITY | 0.008 | -0.088 | 0.529 | 54 | 3.4 | 3.4 | 26.9 | 3.9 |
| 1534-08-3 | STRAWBERRY FLAVOR INTENSITY | 0.007 | 0.086 | 0.535 | 54 | 0.4 | 0.2 | 26.9 | 3.9 |
| 71-41-0 | STRAWBERRY FLAVOR INTENSITY | 0.007 | 0.084 | 0.548 | 54 | 1.0 | 1.3 | 26.9 | 3.9 |
| 123-66-0 | STRAWBERRY FLAVOR INTENSITY | 0.006 | 0.078 | 0.576 | 54 | 108.2 | 128.0 | 26.9 | 3.9 |
| A* ext | STRAWBERRY FLAVOR INTENSITY | 0.005 | 0.073 | 0.599 | 54 | 36.4 | 3.1 | 26.9 | 3.9 |
| 15111-96-3 | STRAWBERRY FLAVOR INTENSITY | 0.005 | -0.071 | 0.608 | 54 | 1.2 | 1.2 | 26.9 | 3.9 |
| 106-32-1 | STRAWBERRY FLAVOR INTENSITY | 0.005 | -0.070 | 0.616 | 54 | 2.2 | 3.0 | 26.9 | 3.9 |
| 1191-16-8 | STRAWBERRY FLAVOR INTENSITY | 0.004 | -0.064 | 0.646 | 54 | 5.5 | 7.0 | 26.9 | 3.9 |
| 75-85-4 | STRAWBERRY FLAVOR INTENSITY | 0.004 | 0.059 | 0.670 | 54 | 3.9 | 2.2 | 26.9 | 3.9 |
| 105-37-3 | STRAWBERRY FLAVOR INTENSITY | 0.002 | -0.049 | 0.723 | 54 | 10.1 | 14.1 | 26.9 | 3.9 |
| 108-10-1 | STRAWBERRY FLAVOR INTENSITY | 0.002 | -0.048 | 0.729 | 54 | 1.5 | 2.6 | 26.9 | 3.9 |
| 140-11-4 | STRAWBERRY FLAVOR INTENSITY | 0.002 | 0.048 | 0.729 | 54 | 11.1 | 8.6 | 26.9 | 3.9 |
| FORCE | STRAWBERRY FLAVOR INTENSITY | 0.002 | -0.048 | 0.730 | 54 | 0.6 | 0.2 | 26.9 | 3.9 |
| 111-27-3 | STRAWBERRY FLAVOR INTENSITY | 0.002 | -0.045 | 0.744 | 54 | 45.5 | 94.6 | 26.9 | 3.9 |
| 2497-18-9 | STRAWBERRY FLAVOR INTENSITY | 0.002 | 0.045 | 0.746 | 54 | 24.9 | 21.5 | 26.9 | 3.9 |
| 624-41-9 | STRAWBERRY FLAVOR INTENSITY | 0.002 | 0.045 | 0.748 | 54 | 18.9 | 18.1 | 26.9 | 3.9 |
| 96-04-8 | STRAWBERRY FLAVOR INTENSITY | 0.002 | 0.040 | 0.776 | 54 | 3.1 | 8.0 | 26.9 | 3.9 |
| 109-60-4 | STRAWBERRY FLAVOR INTENSITY | 0.001 | -0.037 | 0.792 | 54 | 3.8 | 2.7 | 26.9 | 3.9 |
| L* ext | STRAWBERRY FLAVOR INTENSITY | 0.001 | 0.034 | 0.809 | 54 | 33.6 | 2.6 | 26.9 | 3.9 |
| 123-92-2 | STRAWBERRY FLAVOR INTENSITY | 0.001 | 0.032 | 0.819 | 54 | 23.0 | 21.5 | 26.9 | 3.9 |
| 55514-48-2 | STRAWBERRY FLAVOR INTENSITY | 0.000 | -0.020 | 0.884 | 54 | 0.5 | 0.5 | 26.9 | 3.9 |
| MALIC ACID | STRAWBERRY FLAVOR INTENSITY | 0.000 | 0.015 | 0.914 | 54 | 212.4 | 51.6 | 26.9 | 3.9 |
| 928-95-0 | STRAWBERRY FLAVOR INTENSITY | 0.000 | -0.010 | 0.943 | 54 | 66.8 | 61.7 | 26.9 | 3.9 |
| 103-09-3 | STRAWBERRY FLAVOR INTENSITY | 0.000 | 0.006 | 0.968 | 54 | 3.0 | 1.1 | 26.9 | 3.9 |
| 1576-95-0 | STRAWBERRY FLAVOR INTENSITY | 0.000 | -0.003 | 0.983 | 54 | 2.1 | 2.0 | 26.9 | 3.9 |

Regression of chemical and physical measures of fruit (X) to panel responses (Y). Coefficient of determination (R^2^), correlation coefficient, p-value, sample size (n), mean and standard deviation of X and Y derived from bivariate fit in JMP 8.
