# Supplementary material for: Strawberry Flavor: Diverse Chemical Compositions, a Seasonal Influence, and Effects on Sensory Perception
Source: PLoS One. 2014 Feb 11;9(2):e88446. doi: 10.1371/journal.pone.0088446 (PMC3921181; doi:10.1371/journal.pone.0088446)
Supplement: Table S5 — Multiple regression for identification of sweetness enhancing volatiles. Individual volatile compound concentrations are regressed against perceived sweetness intensity independent of effect from glucose, fructose, or sucrose, separately. Thirty compounds (*) (α = 0.05) were found to enhance intensity of sweetness independent of at least one of the three sugars. Six compounds (bold) were found to significantly enhance intensity of sweetness independent of all three sugars. (DOCX) [file pone.0088446.s008.docx]

**Table S5. Multiple regression for identification of sweetness enhancing volatiles.**

| **CAS #** | **FRUCTOSE t RATIO** | **FRUCTOSE *p*-VALUE** |  | **SUCROSE t RATIO** | **SUCROSE *p*-VALUE** |  | **GLUCOSE t RATIO** | **GLUCOSE *p*-VALUE** |  |
| --- | --- | --- | --- | --- | --- | --- | --- | --- | --- |
| **1629-58-9** | **5.097** | **0** | ***** | **2.41** | **0.02** | ***** | **4.696** | **0** | ***** |
| 1576-87-0 | 4.566 | 0 | * | 1.024 | 0.311 |  | 4.301 | 0 | * |
| 1576-86-9 | 4.16 | 0 | * | 0.935 | 0.354 |  | 3.915 | 0 | * |
| **2305-05-7** | **3.933** | **0** | ***** | **2.784** | **0.008** | ***** | **3.549** | **0.001** | ***** |
| 3913-81-3 | 3.694 | 0.001 | * | 1.411 | 0.164 |  | 3.494 | 0.001 | * |
| 124-19-6 | 3.696 | 0.001 | * | 0.226 | 0.822 |  | 3.402 | 0.001 | * |
| 6728-26-3 | 3.349 | 0.002 | * | -0.816 | 0.418 |  | 3.314 | 0.002 | * |
| 591-78-6 | 2.807 | 0.007 | * | 0.767 | 0.447 |  | 2.788 | 0.007 | * |
| 5881-17-4 | 2.894 | 0.006 | * | 0.608 | 0.546 |  | 2.662 | 0.01 | * |
| **540-18-1** | **2.71** | **0.009** | ***** | **2.292** | **0.026** | ***** | **2.515** | **0.015** | ***** |
| **2639-63-6** | **2.865** | **0.006** | ***** | **2.892** | **0.006** | ***** | **2.512** | **0.015** | ***** |
| 105-54-4 | 2.533 | 0.014 | * | 0.034 | 0.973 |  | 2.493 | 0.016 | * |
| 564-94-3 | 2.588 | 0.013 | * | -1.322 | 0.192 |  | 2.455 | 0.018 | * |
| 111-71-7 | 2.599 | 0.012 | * | 1.342 | 0.186 |  | 2.283 | 0.027 | * |
| 4077-47-8 | 2.414 | 0.019 | * | 0.299 | 0.766 |  | 2.185 | 0.034 | * |
| 110-93-0 | 2.527 | 0.015 | * | 1.43 | 0.159 |  | 2.165 | 0.035 | * |
| 638-11-9 | 2.311 | 0.025 | * | 1.256 | 0.215 |  | 2.14 | 0.037 | * |
| **142-92-7** | **2.346** | **0.023** | ***** | **2.943** | **0.005** | ***** | **2.096** | **0.041** | ***** |
| **60415-61-4** | **2.309** | **0.025** | ***** | **2.119** | **0.039** | ***** | **2.062** | **0.044** | ***** |
| 116-53-0 | 2.01 | 0.05 | * | -0.286 | 0.776 |  | 2.035 | 0.047 | * |
| 123-86-4 | 2.179 | 0.034 | * | 1.147 | 0.257 |  | 2.008 | 0.05 | * |
| 7452-79-1 | 1.959 | 0.056 |  | -0.785 | 0.436 |  | 1.993 | 0.052 | * |
| 109-21-7 | 2.181 | 0.034 | * | 1.65 | 0.105 |  | 1.961 | 0.055 |  |
| 109-19-3 | 2.005 | 0.05 | * | 1.662 | 0.103 |  | 1.954 | 0.056 |  |
| 616-25-1 | 1.773 | 0.082 |  | 0.795 | 0.43 |  | 1.628 | 0.11 |  |
| 5454-09-1 | 1.804 | 0.077 |  | 2.085 | 0.042 | * | 1.579 | 0.12 |  |
| 96-22-0 | 1.82 | 0.075 |  | 0.912 | 0.366 |  | 1.576 | 0.121 |  |
| 5989-33-3 | 1.869 | 0.067 |  | 1.953 | 0.056 |  | 1.561 | 0.125 |  |
| 2548-87-0 | 1.76 | 0.084 |  | 0.067 | 0.947 |  | 1.509 | 0.138 |  |
| 623-42-7 | 1.455 | 0.152 |  | 0.419 | 0.677 |  | 1.452 | 0.153 |  |
| 29674-47-3 | 1.339 | 0.187 |  | 0.035 | 0.972 |  | 1.362 | 0.179 |  |
| 53398-83-7 | 1.482 | 0.144 |  | 2.556 | 0.014 | * | 1.283 | 0.205 |  |
| 40716-66-3 | 1.521 | 0.134 |  | 1 | 0.322 |  | 1.202 | 0.235 |  |
| 66-25-1 | 1.229 | 0.225 |  | 0.21 | 0.835 |  | 1.178 | 0.244 |  |
| 104-76-7 | 1.189 | 0.24 |  | 2.046 | 0.046 | * | 0.982 | 0.331 |  |
| 556-24-1 | 0.732 | 0.468 |  | 0.972 | 0.336 |  | 0.88 | 0.383 |  |
| 706-14-9 | 1.247 | 0.218 |  | 1.65 | 0.105 |  | 0.879 | 0.384 |  |
| 110-39-4 | 0.96 | 0.341 |  | 2.645 | 0.011 | * | 0.814 | 0.419 |  |
| 628-63-7 | 0.882 | 0.382 |  | 0.427 | 0.671 |  | 0.749 | 0.457 |  |
| 78-70-6 | 0.872 | 0.387 |  | -0.1 | 0.921 |  | 0.7 | 0.487 |  |
| 124-13-0 | 0.871 | 0.388 |  | 0.169 | 0.866 |  | 0.685 | 0.497 |  |
| 75-85-4 | 0.736 | 0.465 |  | -0.306 | 0.761 |  | 0.667 | 0.508 |  |
| 110-43-0 | 1.054 | 0.297 |  | 1.899 | 0.063 |  | 0.657 | 0.514 |  |
| 105-66-8 | 0.88 | 0.383 |  | 2.421 | 0.019 | * | 0.638 | 0.526 |  |
| 623-43-8 | 0.248 | 0.805 |  | -1.396 | 0.169 |  | 0.54 | 0.592 |  |
| 1534-08-3 | 0.488 | 0.628 |  | 1.035 | 0.305 |  | 0.429 | 0.67 |  |
| 71-41-0 | 0.448 | 0.656 |  | 1.938 | 0.058 |  | 0.296 | 0.769 |  |
| 10522-34-6 | 0.536 | 0.594 |  | 2.049 | 0.046 | * | 0.288 | 0.775 |  |
| 112-14-1 | 0.413 | 0.681 |  | 2.292 | 0.026 | * | 0.284 | 0.777 |  |
| 4887-30-3 | 0.392 | 0.697 |  | 2.71 | 0.009 | * | 0.254 | 0.801 |  |
| 7786-58-5 | 0.362 | 0.718 |  | 2.027 | 0.048 | * | 0.227 | 0.821 |  |
| 103-09-3 | 0.172 | 0.864 |  | -1.053 | 0.297 |  | 0.21 | 0.835 |  |
| 134-20-3 | -0.009 | 0.993 |  | 1.386 | 0.172 |  | 0.2 | 0.842 |  |
| 15111-96-3 | 0.23 | 0.819 |  | 0.927 | 0.358 |  | 0.192 | 0.849 |  |
| 110-38-3 | 0.281 | 0.78 |  | 2.621 | 0.012 | * | 0.055 | 0.956 |  |
| 96-04-8 | 0.142 | 0.887 |  | -0.646 | 0.521 |  | 0.002 | 0.998 |  |
| 123-66-0 | 0.066 | 0.948 |  | 1.064 | 0.292 |  | -0.001 | 0.999 |  |
| 2311-46-8 | 0.238 | 0.813 |  | 2.211 | 0.032 | * | -0.058 | 0.954 |  |
| 29811-50-5 | 0.016 | 0.987 |  | 0.358 | 0.722 |  | -0.098 | 0.922 |  |
| 1576-95-0 | -0.186 | 0.853 |  | -0.304 | 0.762 |  | -0.231 | 0.819 |  |
| 2497-18-9 | -0.313 | 0.756 |  | -0.056 | 0.955 |  | -0.331 | 0.742 |  |
| 20664-46-4 | -0.17 | 0.865 |  | 1.383 | 0.173 |  | -0.395 | 0.694 |  |
| 624-24-8 | -0.406 | 0.686 |  | 0.662 | 0.511 |  | -0.416 | 0.679 |  |
| 589-38-8 | -0.217 | 0.829 |  | 0.592 | 0.557 |  | -0.427 | 0.671 |  |
| 109-60-4 | -0.491 | 0.626 |  | -0.224 | 0.823 |  | -0.454 | 0.652 |  |
| 821-55-6 | -0.267 | 0.791 |  | 1.192 | 0.239 |  | -0.467 | 0.642 |  |
| 624-41-9 | -0.433 | 0.667 |  | -1.323 | 0.192 |  | -0.494 | 0.624 |  |
| 140-11-4 | -0.361 | 0.72 |  | -1.357 | 0.181 |  | -0.513 | 0.61 |  |
| 1191-16-8 | -0.581 | 0.564 |  | -2.268 | 0.028 | * | -0.529 | 0.599 |  |
| 106-70-7 | -0.321 | 0.75 |  | 0.802 | 0.426 |  | -0.531 | 0.598 |  |
| 110-62-3 | -0.572 | 0.57 |  | 0.997 | 0.323 |  | -0.568 | 0.573 |  |
| 105-37-3 | -0.623 | 0.536 |  | 1.964 | 0.055 |  | -0.674 | 0.503 |  |
| 55514-48-2 | -0.644 | 0.523 |  | -0.889 | 0.378 |  | -0.675 | 0.502 |  |
| 123-92-2 | -0.725 | 0.472 |  | 0.108 | 0.914 |  | -0.771 | 0.444 |  |
| 539-82-2 | -0.48 | 0.633 |  | 2.273 | 0.027 | * | -0.802 | 0.426 |  |
| 2432-51-1 | -0.956 | 0.344 |  | 2.033 | 0.047 | * | -1.092 | 0.28 |  |
| 128-37-0 | -0.856 | 0.396 |  | 0.482 | 0.632 |  | -1.17 | 0.247 |  |
| 111-27-3 | -1.253 | 0.216 |  | -0.542 | 0.59 |  | -1.481 | 0.145 |  |
| 928-95-0 | -1.842 | 0.071 |  | -0.516 | 0.608 |  | -1.521 | 0.134 |  |
| 108-10-1 | -2.185 | 0.034 | * | 0.898 | 0.374 |  | -2.138 | 0.037 | * |
| 106-32-1 | -1.436 | 0.157 |  | 0.193 | 0.848 |  | -2.36 | 0.022 | * |

Individual volatile compound concentrations are regressed against perceived sweetness intensity independent of effect from glucose, fructose, or sucrose, separately. Thirty compounds (*) (α = 0.05) were found to enhance intensity of sweetness independent of at least one of the three sugars. Six compounds (bold) were found to significantly enhance intensity of sweetness independent of all three sugars.
